# tRF‐5004b Enriched Secretory Autophagosomes Induce Endothelial Cell Activation to Drive Acute Respiratory Distress Syndrome

**DOI:** 10.1002/advs.202503014

**Published:** 2025-06-20

**Authors:** Xing‐xing Zhu, Han Li, Shuang‐feng Zi, Ying Tang, Na Liu, Ke‐xin Li, Shi‐ke Geng, Hui‐qing Lu, Zhi‐kang Xie, Xin‐yi Xu, Yi‐ting Wei, Li‐xin Wang, Tao Liu, Jie Chao, Yi Yang, Hai‐bo Qiu, Wei Huang, Ling Liu

**Affiliations:** ^1^ Jiangsu Provincial Key Laboratory of Critical Care Medicine Department of Critical Care Medicine Zhongda Hospital School of Medicine Southeast University Nanjing Jiangsu 210009 China; ^2^ Department of Microbiology and Immunology School of Medicine Southeast University Nanjing Jiangsu 210009 China; ^3^ Department of Physiology School of Medicine Southeast University Nanjing Jiangsu 210009 China

**Keywords:** ARDS, endothelial cells, secretory autophagosomes, tRNA‐derived small RNAs

## Abstract

Acute respiratory distress syndrome (ARDS) is an acute inflammatory lung injury for which effective therapeutic agents are lacking. Excessive endothelial cell (EC) activation is a critical trigger of inflammation. Extracellular vesicles (EVs) are increasingly recognized as prominent regulators of inflammatory responses. The previous study identified secretory autophagosomes (SAPs), a novel class of EVs, as a prognostic marker in ARDS, raising questions of whether and how they are involved in the pathogenesis of ARDS. Here, it is shown that inflamed macrophage‐derived SAPs (MSAPs) exacerbate lung injury by weakening the role of ECs as gatekeepers of immune cell transport within the lung. Bioinformatics and functional studies reveal that tRF‐5004b is a key molecule of MSAPs in mediating endothelial activation. Mechanically, tRF‐5004b directly interacts with the nuclear transporter KPNA2, thereby facilitating the association between KPNA2 and the transcription factor p65. This interaction enhances p65 nuclear translocation, a process implicated in EC activation. Additionally, the level of tRF‐5004b is positively correlated with the severity of ARDS, and patients with high tRF‐5004b levels have a poor prognosis. Overall, it is found that tRF‐5004b‐enriched SAPs induce acute lung injury by promoting p65 nuclear translocation to activate ECs, suggesting that tRF‐5004b may be a novel therapeutic target for ARDS.

## Graphical abstract

Acute respiratory distress syndrome (ARDS) is a severe lung condition with limited treatments, often triggered by overactive endothelial cells (ECs). It is identified that macrophage‐derived secretory autophagosomes (MSAPs) exacerbate lung damage by activating EC function. Specifically, tRF‐5004b within MSAPs interacts with KPNA2, facilitating the nuclear translocation of p65, thereby activating ECs. tRF‐5004b levels effectively predict ARDS severity and 28‐day patient outcomes.

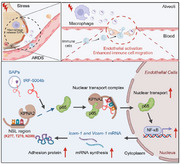

## 1 Introduction

Acute respiratory distress syndrome (ARDS) is a common, often fatal, inflammatory lung injury with an in‐hospital mortality rate reaching 40% and above.^[^ ^1^ ^]^ To date, all pharmacotherapies developed to treat ARDS have failed during clinical trials, highlighting the urgent need to elucidate the pathophysiology of this syndrome, with the aim of identifying new effective drug targets.^[^ ^2^ ^]^ Endothelial cell (EC) activation is a hallmark of ARDS and is the first step of the inflammatory response to lung injury;^[^ ^3^ ^]^ this is due to the expression of adhesion molecules on the surface of ECs to instruct the entry of numerous myeloid cells into lesion sites.^[^ ^4^ ^]^ These myeloid cells, in turn, release excessive inflammatory cytokines to trigger a “cytokine storm” and subsequent damage to the lung tissue. A correlation between the degree of endothelial activation and both prolonged mechanical ventilation duration and increased mortality in ARDS patients has been reported in clinical studies.^[^ ^5^ ^]^ While adhesion molecules and signaling pathways involved in the process of endothelial activation have been extensively studied,^[^ ^6^ ^]^ the potential triggers for EC activation in pulmonary ARDS remain insufficiently explored. Thus, a better understanding of the factors governing EC activation in pulmonary ARDS is imperative for designing new therapeutic interventions.

Recently, emerging evidences have suggested that extracellular vesicles (EVs) are a predominant mechanism of immunomodulation during inflammation progression.^[^ ^7^ ^]^ Studies have demonstrated that EVs facilitate the activation of ECs^[^ ^8^ ^]^ and contribute to the development of ARDS.^[^ ^9^ ^]^ Secretory autophagosomes (SAPs), a subset of EVs characterized by a unique marker, represent a newly attractive mechanism for regulating the course of the immune response.^[^ ^10^ ^]^ Our previous study revealed that the percentage of SAPs in the BALF is elevated in patients with ARDS and is associated with the risk of death in ARDS patients, with macrophages being the primary source of SAPs.^[^ ^11^ ^]^ However, the role of macrophage‐derived SAPs (MSAPs) in the development of ARDS through the activation of ECs has not been thoroughly investigated. Gaining insights into the complex interactions that occur between MSAPs and the activation of ECs may thus lead to novel therapeutic approaches.

EVs, including SAPs, mainly modulate the phenotype and function of recipient cells by transferring bioactive cargoes, such as a wide array of noncoding RNAs. tRNA‐derived small RNAs (tsRNAs), which are derived from mature tRNA or tRNA precursors and exist in various organisms,^[^ ^12^ ^]^ represent an important and increasingly valued type of small non‐coding RNA. These tsRNAs exploit their linear sequences and newly arranged 3D structures to perform biological functions that differ from those of tRNA and play critical roles in a wide spectrum of human diseases including neurological disorders and cancer.^[^ ^13^ ^]^ For example, 5′tiRNA‐His‐GTG promotes CRC progression by targeting LARS2 and modulating the hippo signaling pathway.^[^ ^14^ ^]^ Moreover, tsRNAs can be encapsulated in EVs and delivered to targeted cells to perform their biological functions. Specific tsRNAs are preferentially abundant in osteoblast‐derived EVs, which are taken up by bone marrow cells to increase cell proliferation and increase the survival rate of septic mice.^[^ ^15^ ^]^ Thus, we questioned whether tsRNAs can also act as inflammatory mediators encapsulated by MSAPs and contribute to EC activation.

In this study, we demonstrated that MSAPs are potent activators of ECs that promote leukocyte adhesion and recruitment, which exacerbates lung injury. tRF‐5004b, enriched in MSAP, was identified as a critical tsRNA for modulating the activation of ECs both in vitro and in vivo. Mechanistically, tRF‐5004b interacts with KPNA2 in ECs to facilitate the nuclear translocation of p65, which is responsible for mediating endothelial activation. Furthermore, the level of tRF‐5004b is positively correlated with the poor prognosis of ARDS patients. Overall, this study sheds light on a previously unappreciated reciprocal interaction between MSAPs and ECs, thereby presenting a promising avenue for therapeutic intervention in ARDS.

## 2 Results

### 2.1 Macrophage‐Derived SAPs Promote Endothelial Cell Activation and Lead to Acute Lung Injury

**Figure 1:**
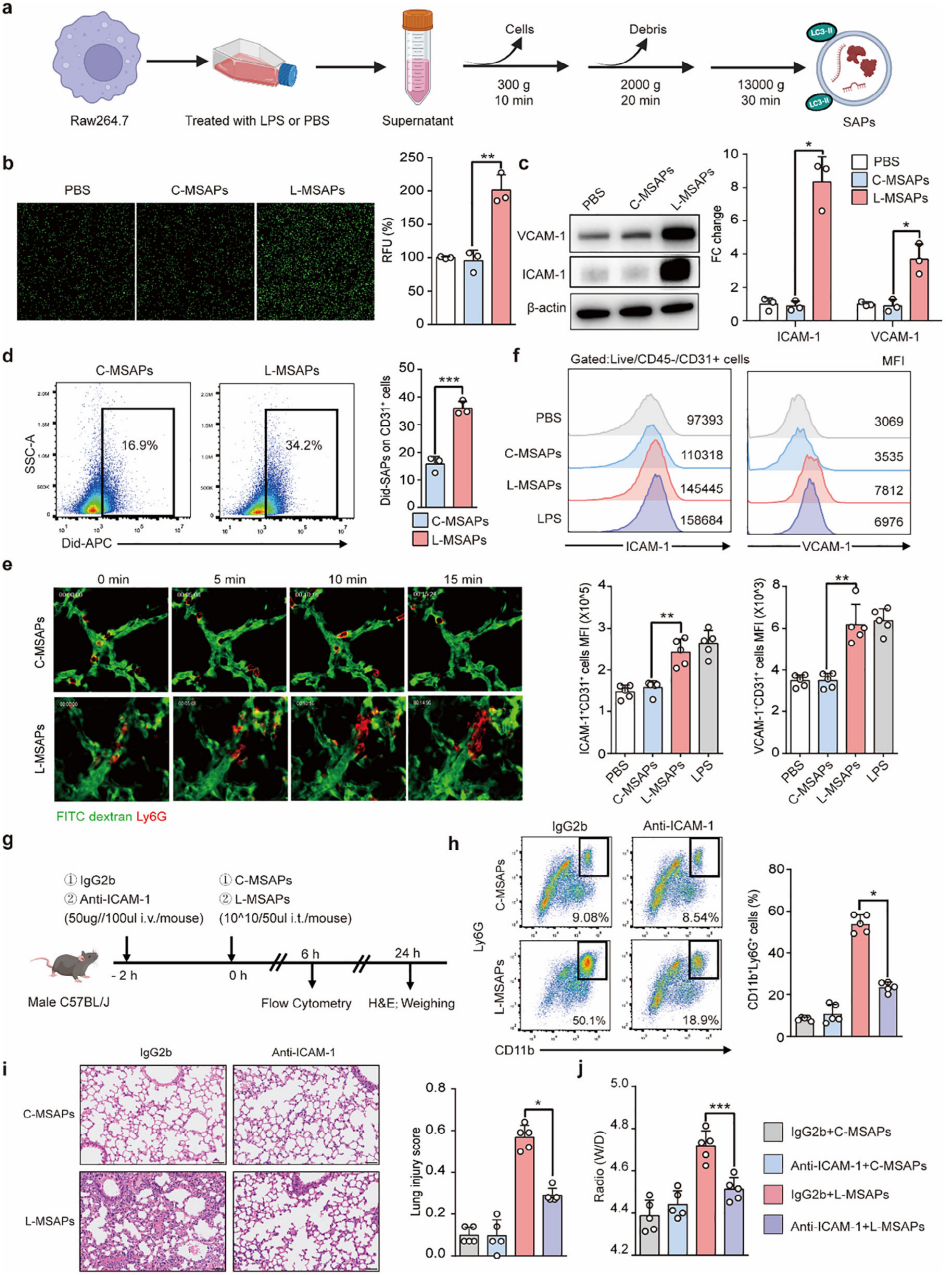
L‐MSAPs target ECs to adhere more neutrophils and cause ALI. a) Schematic diagram of the process of extracting SAPs from the macrophage. b) Representative image of endothelial adhesion PMNs and fluorescence intensity after L‐MSAPs treatment compared with the control (*n* = 3). c) Western blot assay to measure the expression of adhesion molecule (ICAM‐1 and VCAM‐1) in ECs incubated with PBS, C‐MSAPs, or L‐MSAPs (*n* = 3). d) Quantitative analysis of the amount of Did‐C‐MSAPs or L‐MSAPs taken up by ECs in the lung (*n* = 3). e) Time‐lapse confocal images showing an endothelial adhesion neutrophil event after C‐MSAPs or L‐MSAPs treatment (Movies S1 and S2, Supporting Information). f) Flow cytometry analysis of adhesion molecule (ICAM‐1 and VCAM‐1) on ECs in the lung (*n* = 5). g) Schematic of anti‐ICAM‐1 administration and tissue harvest. h) Flow cytometric analysis of pulmonary PMN proportion in L‐MSAPs injected mice pretreated with anti‐ICAM‐1 at 6 h (*n *= 5). i) Representative images of H&E‐stained lung sections in mice and lung injury score 24 h after treatment (*n* = 5). Scale bar: 50 µm. j) W/D ratio of lung tissues of mice with indicated intervention after 24 h (*n* = 5). Statistics: unpaired two‐tailed *t*‐test or two‐tailed Mann–Whitney *U*‐test in (b,d); one‐way ANOVA with Tukey's multiple‐comparisons test or Kruskal–Wallis test with Dunn's multiple comparison test in (c,f,h–j). Data are represented as mean ± SEM. *p < 0.05, **p < 0.01, and ***p < 0.001.

To explore the function of SAPs in lung injury, we first examined the level of SAPs in ARDS and found that SAP levels were higher in bronchoalveolar lavage fluid (BALF) of ARDS model mice than in the BALF of control mice (Figure S1a, Supporting Information). Notably, consistent with our previous study showing that the proportion of MSAPs was dramatically elevated in ARDS patients,^[^ ^11^ ^]^ MSAPs constituted more than 50% of the total SAP population in the BALF, with ARDS mice exhibiting a greater proportion of MSAPs (Figure S1b, Supporting Information). Thus, we isolated MSAPs by differential centrifugation from the supernatant of the RAW264.7 mouse macrophage line for subsequent experiments. MSAPs were verified by western blot, flow cytometry, transmission electron microscopy (TEM), and nanoparticle tracking analysis (NTA). Western blot and flow cytometry analyses confirmed the presence of the characteristic SAP protein LC3 (Figure S1c,d, Supporting Information). MSAPs were physically homogenous with sizes peaking at 214.2 nm in diameter as determined by TEM and NTA (Figure S1e,f, Supporting Information). To closely mimic the ARDS environment, L‐MSAPs were generated by stimulating macrophages with lipopolysaccharide (LPS), while C‐MSAPs, the control, were produced by treating macrophages with PBS (**Figure** **1**a). The mice were given an intratracheal instillation of PBS, C‐MSAPs, L‐MSAPs, or LPS. The lung tissue in the C‐MSAP group was not damaged, similar to that in the PBS group. In contrast, the lung tissue of the mice that were injected with L‐MSAPs or LPS exhibited substantial inflammatory cell infiltration, fluid exudation, and alveolar septal thickening, and the lung injury score was significantly greater in the L‐MSAP group than in the C‐MSAP group (Figure S1g, Supporting Information). Compared with those in the C‐MSAP group, the lung wet‐to‐dry weight ratio (Figure S1h, Supporting Information) and the expression of proinflammatory cytokines (Figure S1i, Supporting Information) in the lung tissues were significantly increased in the L‐MSAP‐treated group. Overall, these findings indicate that L‐MSAPs are key mediators of lung injury.

We employed CellChat analysis based on single‐cell RNA sequencing derived from mouse lung tissues subjected to sham or acute lung injury to decipher if there was direct communication between macrophages and ECs. Among the interactions between macrophages and parenchymal cells, only communication directed toward ECs was significantly enhanced in the disease state (Figure S2, Supporting Information). It has been well‐established that EVs are positioned as prominent regulators of immunomodulation by delivering and exchanging cellular contents between effector and target cells during inflammation progression. Hence, we hypothesized that SAP serves as a crucial mediator in the communication between macrophages and ECs. To test this hypothesis, we assessed the uptake of MSAPs by ECs in vitro. As shown in Figure S3 (Supporting Information), the number of DiD‐labelled L‐MSAPs (red fluorescence) in ECs progressively increased with increasing coincubation duration. These results suggest that macrophages may communicate with ECs via SAPs.

Next, we investigated how MSAPs regulate endothelial functions. Bulk RNA sequencing showed that a total of 189 endothelial genes were significantly upregulated upon L‐MSAP treatment (Figure S4a, Supporting Information). Gene Ontology (GO) analysis of the upregulated genes showed enrichment of terms related to cell adhesion (Figure S4b, Supporting Information). Consistently, GO analysis of the differentially upregulated genes in the ECs of acute lung injury model mice also showed that most of the identified biological processes were related to immune regulation, including leukocyte migration (Figure S5a, Supporting Information). Furthermore, the expression of the endothelial adhesion molecule ICAM‐1, which plays a crucial role in the regulation of leukocyte migration, was significantly elevated in the acute lung injury group (Figure S5b, Supporting Information). To confirm the potential regulatory role of L‐MSAPs in modulating endothelial cell adhesion and subsequent leukocyte migration, ECs were exposed to varying concentrations of L‐MSAPs. While C‐MSAPs had no effect on EC activation, L‐MSAPs induced EC activation and adhesion molecule expression in a dose‐dependent manner (Figure 1b,c; Figure S6a–c, Supporting Information), suggesting that L‐MSAPs act as potential activation factors for ECs.

To evaluate the effects of MSAPs on endothelial cells (ECs) in vivo, we investigated their cellular uptake and function following intratracheal administration. Immunofluorescence analysis revealed PKH26‐labeled MSAPs co‐localizing with pulmonary ECs, demonstrating their capacity for MSAP internalization (Figure S7, Supporting Information). Flow cytometry subsequently confirmed that the proportion of endothelium ingested by DiD‐labelled L‐MSAPs was significantly higher than that ingested by C‐MSAPs (Figure 1d). This finding further supporting the view that macrophages crosstalk with ECs via SAPs is an important event in acute lung injury. To better visualize EC activation in vivo, we conducted confocal intravital microscopy (IVM) of pulmonary vascular images and found that neutrophils slowly rolled and exhibited increased adhesion to the endothelium within a few hours of intratracheal instillation with L‐MSAPs (Figure 1e; Movie S1, Supporting Information). Additionally, flow cytometric analysis of lung tissue revealed an upregulation in the expression of ICAM‐1 and VCAM‐1 in ECs (Figure 1f), along with a significant increase in the number of neutrophils in the lung tissue following L‐MSAP administration (Figure S8a–c, Supporting Information). Moreover, the administration of an anti‐ICAM‐1 antibody effectively reversed the increase in the proportion of neutrophils in the lung tissue and mitigated the severity of lung injury in the mice treated with L‐MSAPs (Figure 1g–j). In addition, SAPs extracted from the BALF of patients with ARDS have been shown to induce the upregulation of ICAM‐1 expression in human vascular endothelial cell line EAhy926 comparison with the control group (Figure S9, Supporting Information). Taken together, these results indicate that L‐MSAPs induce endothelial activation to amplify inflammation and drive lung injury.

### 2.2 MSAP‐Carried tRF‐5004b Mediates Endothelial Activation

EVs modulate the fate of target cells through the transfer of functional molecules. Recent studies have demonstrated that miRNAs and tsRNAs constitute the majority of small RNAs in vesicles secreted by macrophages under inflammatory conditions, accounting for 58% and 23%, respectively.^[^ ^16^ ^]^ To ascertain the function of small RNAs in MSAPs, we conducted a non‐coding transcriptome analysis of both C‐MSAPs and L‐MSAPs. tsRNAs showed more significant changes than miRNAs did (Figure S10a,b, Supporting Information). Remarkably, the knockdown of XPOT, a vital protein for tsRNA biogenesis,^[^ ^17^ ^]^ in macrophages led to a global reduction in tsRNAs (Figure S10c,d, Supporting Information), and compared with control MSAPs, MSAPs derived from XPOT‐knockdown cells attenuated endothelial activation (Figure S10e,f, Supporting Information), highlighting the crucial role of tsRNAs in EC activation.

**Figure 2:**
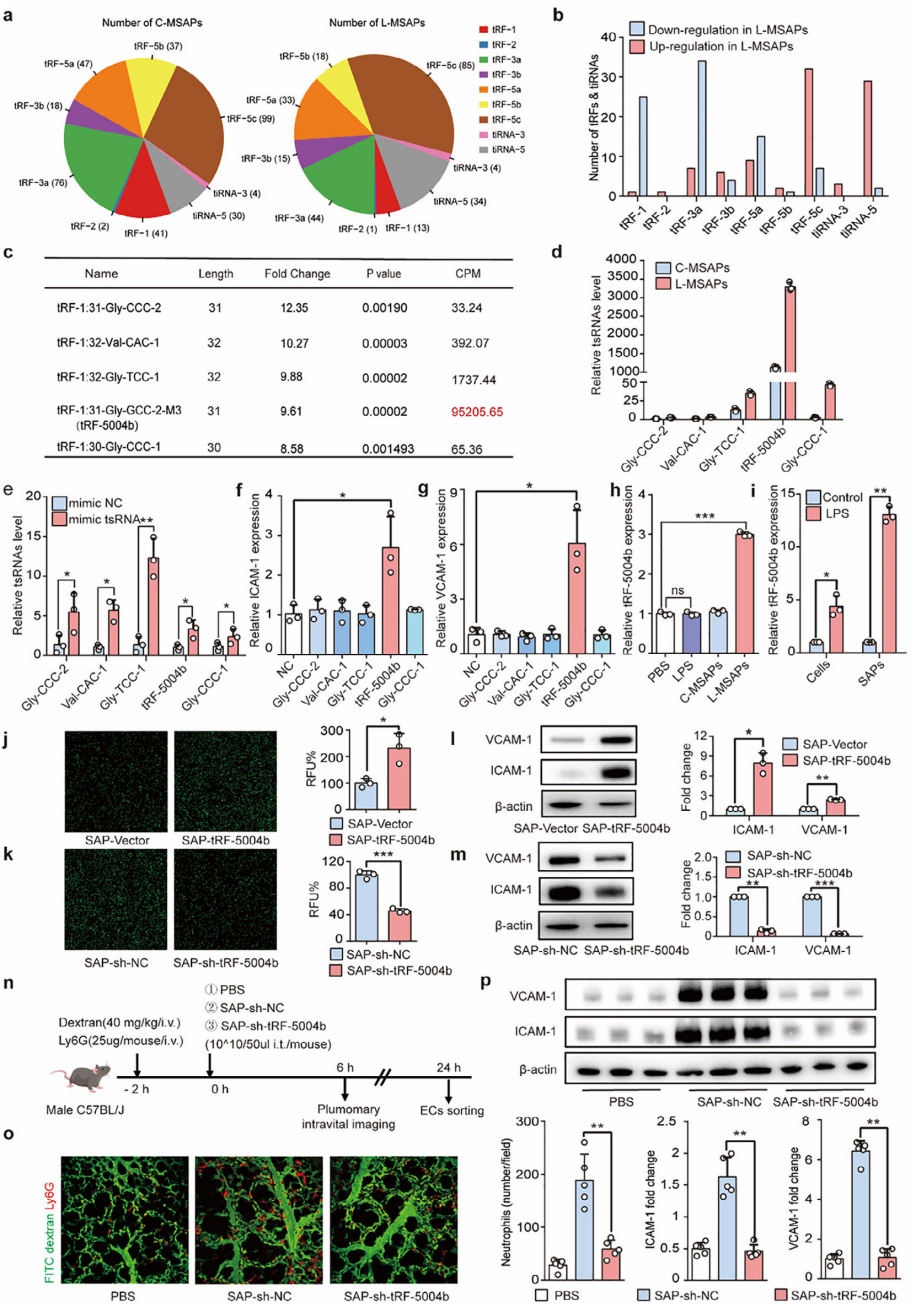
In vitro and in vivo studies showing tRF‐5004b functioned as a pro‐adherent tsRNA in L‐MSAPs to activate ECs. a) The proportions of different types of tsRNA in C‐MSAPs and L‐MSAPs groups. b) The numbers of significantly upregulated and downregulated (set *P* < 0.05) tRFs and tiRNAs were separately calculated in L‐MSAPs, and tRF‐5c was the main upregulated type. c) The five of tRF‐5c with the highest fold change was selected for further function verification. d) The expression levels of the top five tsRNA in L‐MSAPs and C‐MSAPs (*n* = 3). e–g) qRT‐PCR was used to verify the transfection efficiency and detect the expression of endothelial adhesion molecules after transfection of the five indicated tRF‐5c mimics (*n* = 3). h) qRT‐PCR to measure the expression of tRF‐5004b in ECs incubated with PBS, LPS, C‐MSAPs, or L‐MSAPs (*n* = 3). i) qRT‐PCR analysis of tRF‐5004b level in macrophages and MSAPs (*n* = 3). j–m) ECs were pretreated with SAP‐tRF‐5004b or SAP‐sh‐tRF‐5004b and their respective controls for 12 h. The levels of endothelial adhesion to neutrophils were quantified by endothelium–leukocyte adhesion experiment (j,k), and the adhesion molecule (ICAM‐1 and VCAM‐1) expression in ECs was detected by western blotting (l,m) (*n* = 3). n) Schematic showing the administration of SAP‐sh‐tRF‐5004b, intravital imaging, and cell sorting. o) Representative intravital imaging of Ly6G‐labeled PMNs (red) and pulmonary microcirculation (FITC dextran, green). Quantification of PMN infiltration in lungs 6 h after SAP‐sh‐tRF‐5004b treated (one fields of view per mouse, *n *= 5). Scale bar: 50 µm. p) Western blotting analysis of ICAM‐1 and VCAM‐1 in ECs sorted from mice lung tissue (*n* = 5). Statistics: unpaired two‐tailed *t*‐test or two‐tailed Mann–Whitney *U*‐test in (e–m); Kruskal–Wallis test with Dunn's multiple comparison test in (o, p). Data are represented as mean ± SEM. ns, no significance. *p < 0.05, **p < 0.01 and ***p < 0.001.

The proportions of different subtypes of tsRNAs in the C‐MSAP and L‐MSAP groups were further analyzed and tRF‐5c was the predominant subtype (**Figure** **2**a). Notably, tRF‐5c also presented the largest number of upregulated tsRNAs among the L‐MSAPs (Figure 2b). We therefore selected the top five tsRNAs of the tRF‐5c subtype with the greatest fold changes for further functional verification, with tRF‐5004b exhibiting the most significant changes in the C‐MSAP and L‐MSAP groups (Figure 2c,d). Upon the transfection of ECs with five tsRNA mimics respectively, only tRF‐5004b was found to induce the expression of *Icam‐1* and *Vcam‐1* in ECs (Figure 2e–g), indicating that tRF‐5004b may be the key tsRNA in MSAPs that regulates EC activation. Interestingly, while LPS treatment showed no effect on tRF‐5004b expression in ECs, exposure to L‐MSAPs resulted in significant upregulation of tRF‐5004b (Figure 2h). Thus, tRF‐5004b expression in ECs appears to be specifically triggered by MSAPs rather than direct inflammatory stimulation.

tRF‐5004b was markedly upregulated in MSAPs derived from the LPS‐stimulated RAW264.7 mouse macrophage cell line, a phenomenon that was similarly observed in both THP‐1‐derived macrophages and mouse bone marrow‐derived macrophages (Figure 2i; Figure S11a–d, Supporting Information). Thus, we sought to determine whether this increase is partly dependent on the elevated production of tRF‐5004b by macrophages following LPS stimulation. The results from quantitative analysis of the intracellular tRF‐5004b levels showed a marked increase following LPS treatment (Figure 2i). RNA modification is a crucial component of epigenetic regulation and plays a significant role in RNA biogenesis.^[^ ^18^ ^]^ ALKBH3, identified as the first mammalian tRNA demethylase, enhances the cleavage sensitivity of tRNA post‐demethylation, thereby facilitating tsRNA production.^[^ ^19^ ^]^ We found that the expression of ALKBH3 in macrophages was upregulated following stimulation with LPS (Figure S12a, Supporting Information). Furthermore, the knockdown of ALKBH3 via the use of a specific siRNA (si‐ALKBH3) in macrophages resulted in a marked reduction in the levels of several tsRNAs, including tRF‐5004b (Figure S12b–f, Supporting Information). These results imply that macrophages may undergo tRNA demethylation within an inflammatory environment, exerting biological functions through the production of tsRNAs and the secretion of tsRNAs into SAPs.

Recent evidence suggests that the composition of EVs is cell‐specific and regulated by complex mechanisms.^[^ ^20^ ^]^ This implies the existence of a specialized sorting mechanism for packaging tRF‐5004b into SAPs. To investigate the mechanism underlying tRF‐5004b's selective enrichment in SAPs, we performed proteomic analysis in SAPs. This approach identified HNRNPA2B1 as the predominant RNA‐binding proteins (RBPs) with sorting ability, based on its significantly higher peptide spectrum match count compared to other RBPs (Figure S13a, Supporting Information). We confirmed that HNRNPA2B1 can bind to tRF‐5004b by RNA pull down and western blot (Figure S13b, Supporting Information). Subsequently, we employed siRNA techniques to knockdown HNRNPA2B1 in RAW264.7 cells and collected MSAPs secreted by both control and HNRNPA2B1‐knockdown cells. Compared to the control MSAPs, the tRF‐5004b content in MSAPs derived from HNRNPA2B1 knockdown cells was significantly reduced (Figure S13c,d, Supporting Information), indicating that HNRNPA2B1 plays a critical role in the sorting of tRF‐5004b into SAPs. Additionally, the selective loading of miRNAs is facilitated by RBPs that recognize specific short nucleotide sequences, known as “motifs,” within miRNAs.^[^ ^21^ ^]^ Thus, we aimed to elucidate the specific motif responsible for HNRNPA2B1‐mediated sorting of tRF‐5004b. It is reported that AGG/UAG motifs are specifically recognized by HNRNPA2B1.^[^ ^22^ ^]^ Notably, sequence analysis revealed that tRF‐5004b contains a conserved UAG motif. To functionally validate this interaction, we mutated the binding motif UAG to AUC (Mut) and performed a biotinylated probe pull‐down assay. Strikingly, HNRNPA2B1 showed specific bindings to wild‐type but not mutant tRF‐5004b probes (Figure S13e, Supporting Information). Taken together, we concluded that HNRNPA2B1 binds directly to the UAG motif in the tRF‐5004b. In addition, LC3 is reported to be essential for the loading and secretion of some RNA cargo into extracellular vesicles through its interaction with RBPs.^[^ ^23^ ^]^ To investigate whether this mechanism applies to HNRNPA2B1‐mediated RNA sorting, we examined the potential interaction between HNRNPA2B1 and LC3 in LPS‐stimulated RAW264.7 cells. Co‐immunoprecipitation (Co‐IP) assays revealed a specific interaction between HNRNPA2B1 and LC3 (Figure S13f, Supporting Information), suggesting their potential cooperation in SAP cargo sorting. Consequently, these findings imply that proteins HNRNPA2B1 and LC3 may play a primary role in the sorting of tRF‐5004b into SAPs.

To further investigate the biological effects of tRF‐5004b in MSAPs on ECs, overexpression and knockdown models were established in RAW264.7 cells to produce SAPs with high expression of tRF‐5004b (SAP‐tRF‐5004b) and low expression of tRF‐5004b (SAP‐sh‐tRF‐5004b) (Figure S14a–d, Supporting Information). Cell adhesion assays showed that SAP‐tRF‐5004b significantly activated ECs, whereas SAP‐sh‐tRF‐5004b inhibited EC activation (Figure 2j,k). Additionally, SAP‐tRF‐5004b led to the upregulation of ICAM‐1 and VCAM‐1 expression, whereas SAP‐sh‐tRF‐5004b resulted in their downregulation (Figure 2l,m). Next, the mice were intratracheally injected with PBS, SAP‐sh‐NC, or SAP‐sh‐tRF‐5004b (Figure 2n). The decrease in tRF‐5004b levels in MSAPs led to a reduction in the number of neutrophils that adhered to the pulmonary endothelium (Figure 2o). Western blot analysis showed that the protein levels of adhesion molecules (ICAM‐1 and VCAM‐1) were significantly decreased in FACS‐sorted ECs from the SAP‐sh‐tRF‐5004b group (Figure 2p; Figure S15, Supporting Information).

To further investigate whether tRF‐5004b is predominantly produced by macrophages, an ARDS mouse model was employed, and lung tissues were collected 24 h post‐induction. By comparing the expression of tRF‐5004b in different cell types within the lung tissue after flow sorting, we found that tRF‐5004b expression was significantly increased of all cell types in the ARDS group compared to the sham group, with the highest expression level detected in macrophages (Figure S16a,b, Supporting Information). This phenomenon was also observed by Raman spectroscopy of lung tissue sections, where the tRF‐5004b showed a high degree of colocalization with lung macrophages (Figure S16c,d, Supporting Information). These data suggest that during ARDS, there is an upregulation of tRF‐5004b in macrophages, which subsequently secrete tRF‐5004b‐enriched SAPs, leading to the activation of ECs.

### 2.3 tRF‐5004b Activates NF‐κB Signaling at the p65 Level

**Figure 3:**
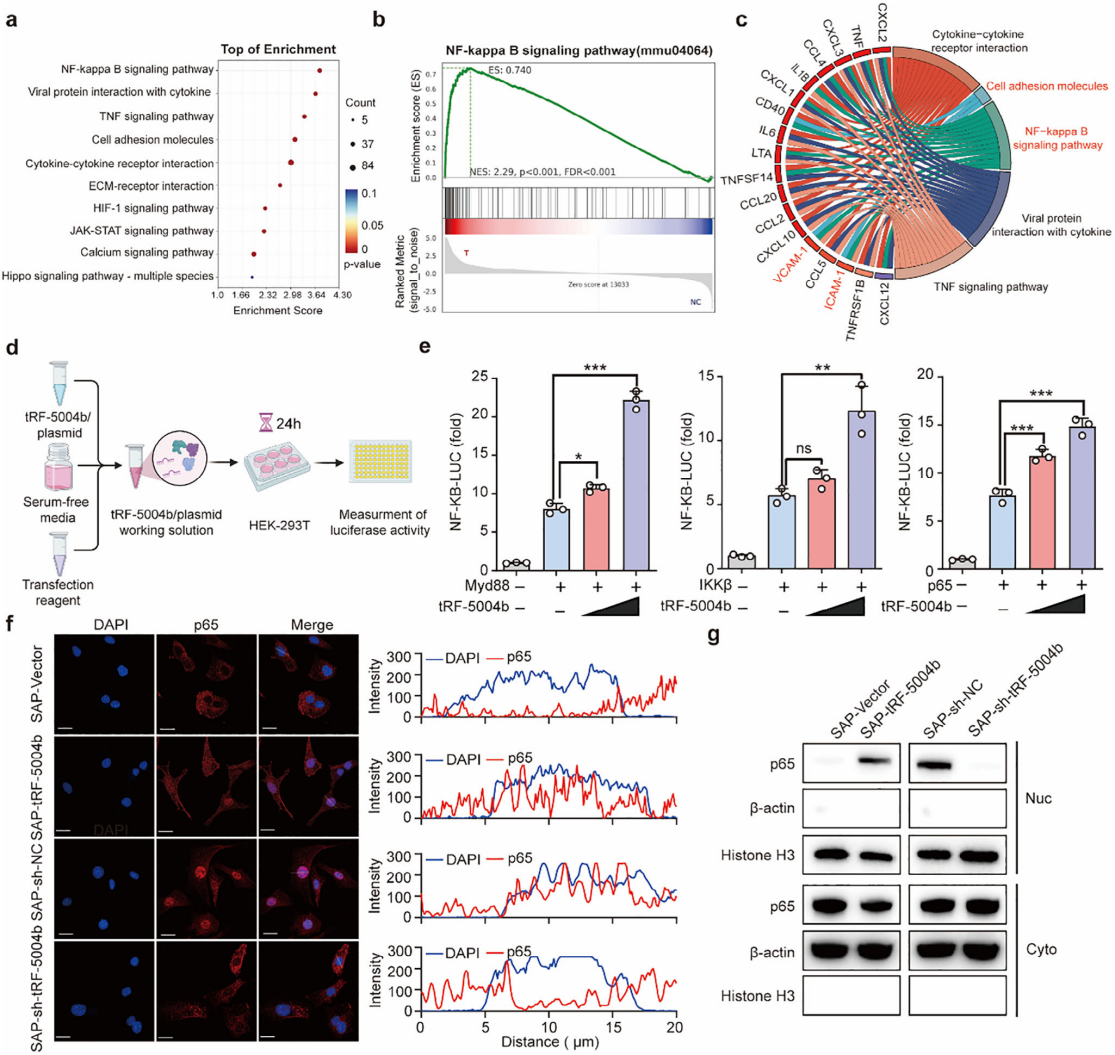
tRF‐5004b promotes NF‐κB signaling activation at the p65 level. a) The top 10 KEGG pathways enriched by differentially expressed genes. Statistical significance was defined as a *p*‐value < 0.05 and |log2FC| > 1. b) GSEA of enrichment for signatures associated with the NF‐κB signaling pathway. c) Visual intersection network of the top five KEGG pathways and the involved genes. d,e) Schematic of co‐transfected tRF‐5004b mimics, pNF‐κB‐Luc, pRL‐TK, and MyD88/IKKβ/p65 plasmids (d), along with NF‐κB activation analysis (e) (*n* = 3). f,g) ECs were treated with SAP‐tRF‐5004b or SAP‐sh‐tRF‐5004b, along with their respective controls. Representative images of immunofluorescence staining showing the translation of p65 (red) in ECs. DAPI: blue, nucleus; scale bar: 20 µm. Line chart of fluorescence signal positioning analysis (f). Western blot analysis of nuclear and cytoplasmic p65 protein levels in ECs (g) (*n* = 3). Statistics: one‐way ANOVA with Tukey's multiple‐comparisons test or Kruskal–Wallis test with Dunn's multiple comparison test in (e). Data are represented as mean ± SEM. ns, no significance. **p < 0.01 and ***p < 0.001.

RNA sequencing (RNA‐seq) profiling was conducted using ECs treated with either the SAP‐Vector or SAP‐tRF‐5004b to investigate the mechanism through which tRF‐5004b modulates endothelial activation. Kyoto Encyclopedia of Genes and Genomes (KEGG) pathway analysis revealed that the NF‐κB signaling pathway was the most significantly enriched pathway among the pathways associated with the upregulated differentially expressed genes (**Figure** **3**a). Gene set enrichment analysis (GSEA) further showed a notable overlap between NF‐κB–dependent gene sets and those regulated by tRF‐5004b, indicating that tRF‐5004b may activate NF‐κB signaling (Figure 3b). Consistent with the tRF‐5004b‐induced endothelial adhesion molecule expression results, numerous downstream genes associated with endothelial adhesion were also significantly enriched in the NF‐κB signaling pathway (Figure 3c).

Next, we sought to determine how tRF‐5004b promotes NF‐κB activation. We conducted cotransfection experiments involving tRF‐5004b and key molecules in the NF‐κB signaling pathway and subsequently assessed the activation of NF‐κB signaling (Figure 3d). As shown in Figure 3e, the overexpression of tRF‐5004b augmented the activation of NF‐κB by MyD88, IKKβ, or p65. Given that p65 is positioned downstream within the MyD88/IKKβ‐mediated NF‐κB signaling pathway, it is highly probable that tRF‐5004b directly modulates p65 to promote NF‐κB signaling activation. The translocation of cytoplasmic NF‐κB p65 to the nucleus represents a critical step in the activation of the NF‐κB pathway. We further investigated the levels of p65 in both the cytoplasmic and nuclear compartments of ECs.  Immunofluorescence staining showed that, while p65 was predominantly localized in the cytoplasm in the control group, there was a notable increase in nuclear p65 levels in the SAP‐tRF‐5004b group (Figure 3f). SAP‐tRF‐5004b markedly increased the nuclear localization of p65, whereas SAP‐sh‐tRF‐5004b impeded this process (Figure 3g). These findings collectively suggest that tRF‐5004b may promote the translocation of p65 into the nucleus, thereby augmenting NF‐κB activation.

### 2.4 tRF‐5004b Induces the Translocation of p65 Protein into the Nucleus in an Importin‐Dependent Manner

**Figure 4:**
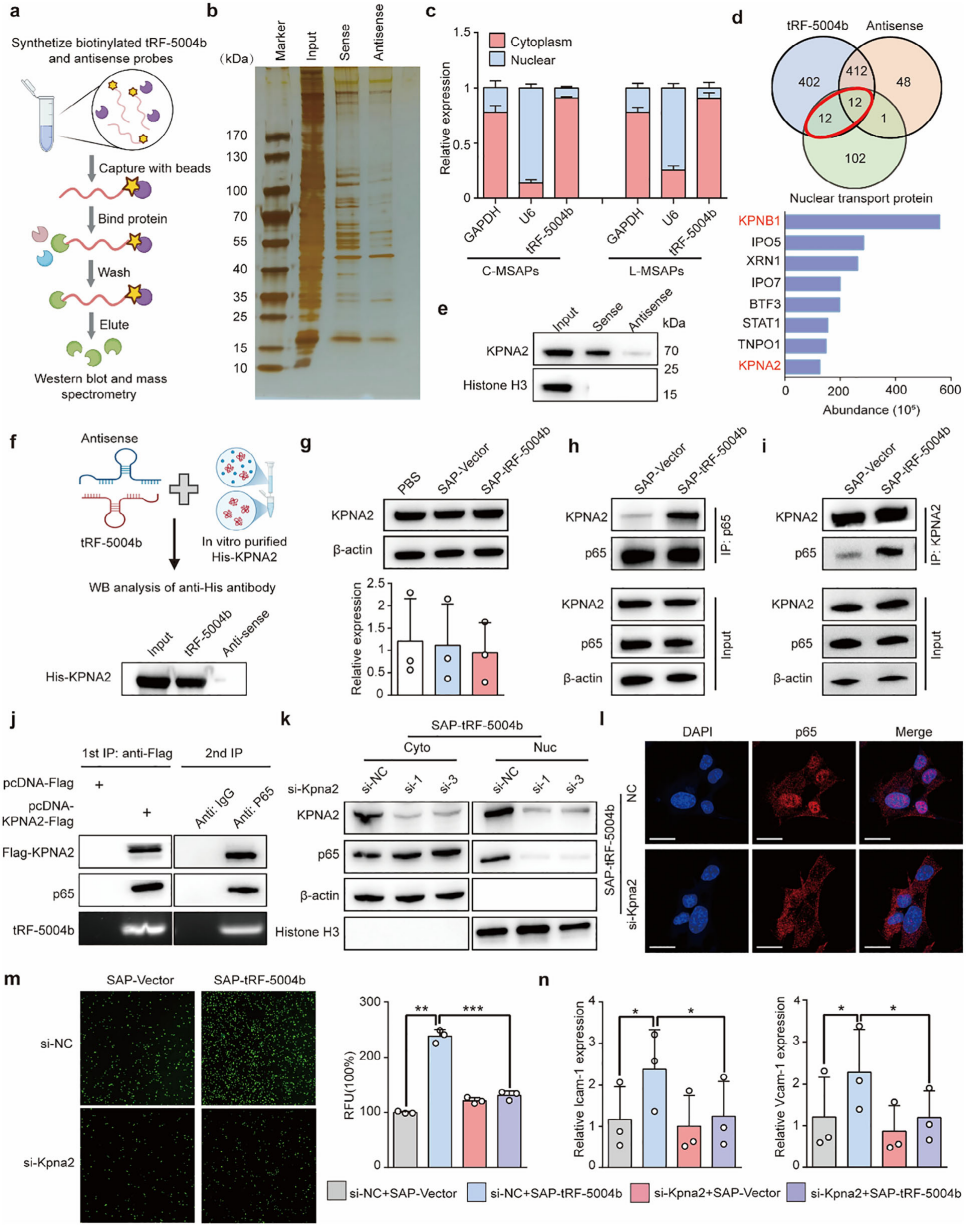
tRF‐5004b directly binds KPNA2 to promote p65 nuclear translocation. a) The workflow of tRF‐5004b pulldown assay. b) Silver staining of proteins pulled down by in vitro transcribed tRF‐5004b and antisense from total protein extracts of EC cells. c) Subcellular localization of tRF‐5004b in ECs, U6, and GAPDH RNAs were used as controls for nuclear, and cytoplasmic fractions, respectively (*n* = 3). d) Venn diagram illustrating the protein expression overlap in the eluted proteins of the tRF‐5004b sense RNA probe and the eluted proteins antisense RNA probe and nuclear transport proteins set. e) KPNA2 was pulled down by tRF‐5004b but not antisense in ECs, as determined by western blotting (*n* = 3). f) In vitro RNA pull‐down assay with purified recombinant His‐KPNA2 and tRF‐5004b (*n* = 3). g) RNA and protein levels of KPNA2 were assessed by PCR and western blot assays, respectively (*n* = 3). h,i) tRF‐5004b promoted the binding between KPNA2 and p65 in ECs (*n* = 3). j) KPNA2, P65, and tRF‐5004b were co‐precipitated with an anti‐Flag antibody in whole‐cell lysates of endothelial cells transfected with Flag‐tagged KPNA2 (left), and after elution with Flag peptide, all were further co‐precipitated with an anti‐P65 antibody in the resultant precipitates(right). k) Western blotting of nuclear and cytoplasmic extracts from si‐KPNA2 or si‐NC ECs treated with SAP‐tRF‐5004b (*n* = 3). Histone H3 and β‐actin were used as nuclear and cytoplasmic controls, respectively. l) Fluorescence staining showing the subcellular distribution of p65 (red) in ECs with KPNA2 knockout (scale bar: 20 µm) (*n* = 1). m) Endothelium–leukocyte adhesion analysis of KPNA2‐knockdown ECs or the NC‐treated ECs stimulated by tRF‐5004b‐SAPs for 12 h (*n* = 3). n) Relative expression of *Icam‐1* and *Vcam‐1* in ECs with KPNA2 knockdown (*n* = 3). Statistics: one‐way ANOVA with Tukey's multiple‐comparisons test or Kruskal–Wallis test with Dunn's multiple comparison test in (m,n). Data are represented as mean ± SEM. *p < 0.05, **p < 0.01, and ***p < 0.001.

To uncover the regulatory mechanism of tRF‐5004b in promoting p65 translocation into the nucleus, we performed an RNA pull‐down assay and mass spectrometry (MS) analysis to identify the potential proteins associated with tRF‐5004b (**Figure** **4**a,b). However, the tRF‐5004b probe‐bound complex did not contain p65, suggesting that the nuclear translocation of p65 induced by tRF‐5004b may be dependent on nuclear transporter proteins. To further elucidate this mechanism, we examined the subcellular localization of tRF‐5004b. qRT‐PCR assays performed following nucleoplasm separation demonstrated that tRF‐5004b is predominantly localized within the cytoplasm (Figure 4c). Interestingly, a subset of nuclear transporter proteins was significantly more abundant in the tRF‐5004b probe group than in the control group (Figure 4d). KPNB1, the protein exhibiting the highest abundance and localized in the cytoplasm,^[^ ^24^ ^]^ was chosen for subsequent verification. The tRF‐5004b probe can precipitate KPNB1 during EC lysis, and KPNB1 knockdown in ECs reduces SAP‐tRF‐5004b‐induced p65 nuclear translocation (Figure S17a–c, Supporting Information). However, tRF‐5004b does not bind to purified His‐tagged KPNB1 proteins in a cell‐free system (Figure S17d, Supporting Information), indicating that an intermediary protein may be involved. In classical nuclear import, cargo initially binds to importin‐α, and its subsequent association with importin‐β facilitates the passage of the complex through the nuclear pore.^[^ ^25^ ^]^ Thus, we speculate that tRF‐5004b may directly bind to KPNA2, the most abundantly expressed importin‐α among the proteins identified by MS. The endogenous interaction of tRF‐5004b and KPNA2 was confirmed in ECs via an RNA pull‐down assay (Figure 4e). Notably, in vitro synthesized tRF‐5004b could bind to the purified His‐KPNA2 protein in a cell‐free system (Figure 4f; Figure S18, Supporting Information), indicating that tRF‐5004b binds directly to KPNA2.

Given that tRF‐5004b does not influence KPNA2 mRNA or protein levels (Figure 4g), we explored the possibility that the interaction between tRF‐5004b and KPNA2 might enhance the binding affinity of KPNA2 for p65. To this end, we performed Co‐IP analysis of KPNA2 and p65 in ECs treated with SAP‐tRF‐5004b or SAP vector, respectively. The results showed that SAP‐tRF‐5004b facilitates the binding of KPNA2 to p65 (Figure 4h,i). To better demonstrate that tRF‐5004b mediates the interaction between KPNA2 and p65 and is essential for p65 nuclear translocation, we performed Flag‐KPNA2 overexpression in ECs via electroporation, followed by two‐step RNA immunoprecipitation (RIP) assays to characterize these molecular interactions. As expected, antibodies against the Flag epitope tag precipitated KPNA2 along with P65 and tRF‐5004b from total protein extracts, whereas in second‐phase immunoprecipitation, anti‐P65 antibodies co‐precipitated KPNA2 and tRF‐5004b (Figure 4j). Strikingly, KPNA2 depletion resulted in a marked reduction in tRF‐5004b‐induced p65 nuclear translocation (Figure 4k,l). Additionally, KPNA2 knockdown diminished the tRF‐5004b‐induced enhancement of adhesion function and the expression of related molecules (ICAM‐1 and VCAM‐1) (Figure 4m,n). These findings suggest that the nuclear translocation of p65 induced by tRF‐5004b enhances the expression of adhesion molecules in an importin‐α KPNA2‐dependent manner.

### 2.5 tRF‐5004b Interacts with the NSL Region of KPNA2 and K277, T278, and N279 are Key Residues Responsible for Binding

**Figure 5:**
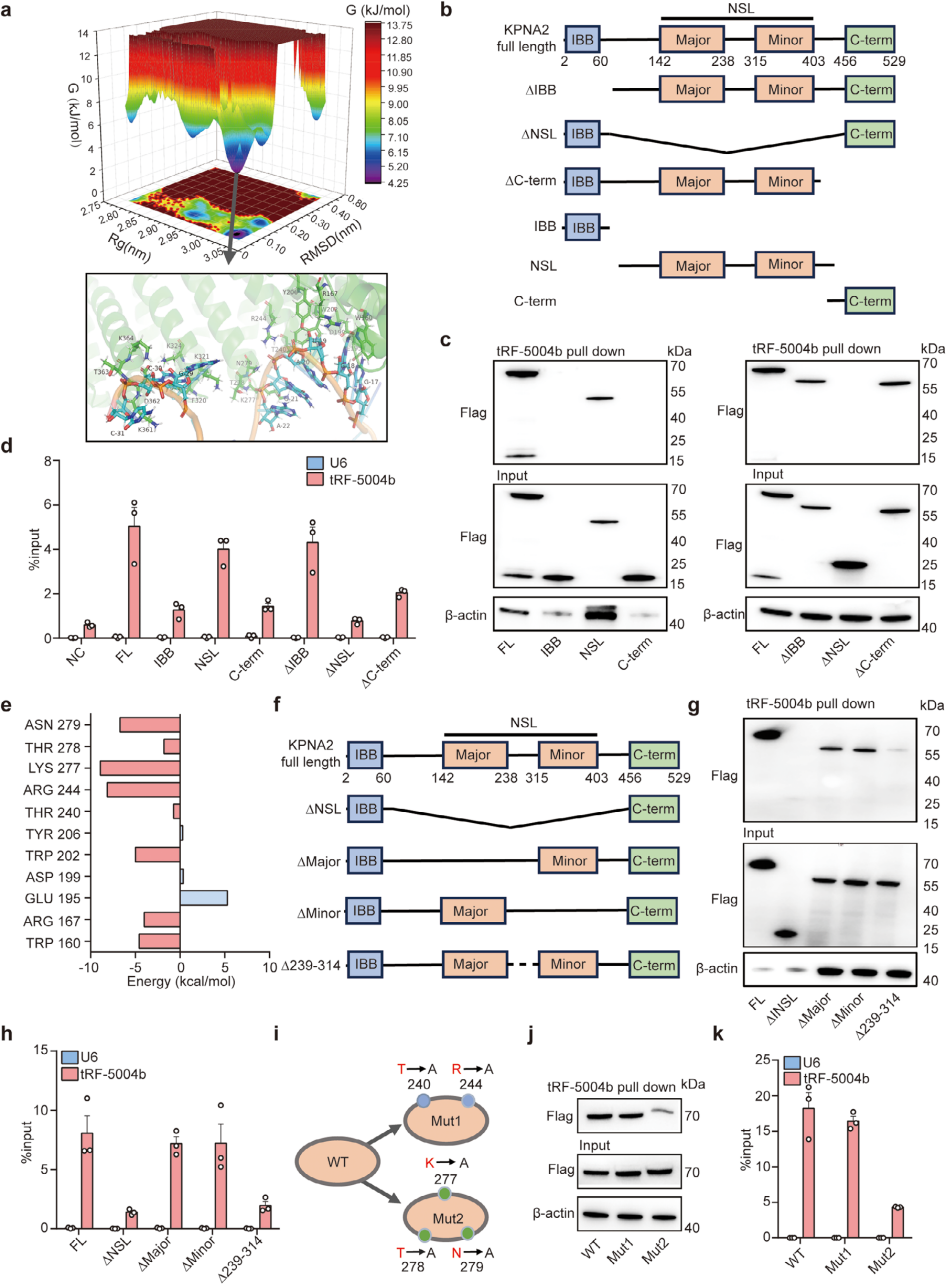
Mapping the binding sites of tRF‐5004b on KPNA2. a) Gibbs free energy landscape of the first two principal components (PCs) generated from MD simulations for the binding between tRF‐5004b and KPNA2, where the dark blue color areas indicate lower energetic conformations. According to the KPNA2 (pale green)‐tRF‐5004b (orange) binding mode extracted from the lowest potential well, the details of the critical molecular interaction between protein‐RNA have been zoomed in. b) Schematic representation of the KPNA2 deletion mutants used in the RNA pull‐down and RIP assays shown in (c,d). c) Western blot of the indicated Flag‐tagged KPNA2 deletion mutants retrieved by in vitro transcribed tRF‐5004b obtained from 293T cell extracts (*n* = 3). d) qRT‐PCR analysis of endogenous tRF‐5004b enriched by the indicated Flag‐tagged KPNA2 deletion mutants in 293T cells (*n* = 3). U6 mRNA served as the negative control. e) The free energy values of binding sites of tRF‐5004b in NSL regions of KPNA2. f) Schematic representation of the KPNA2 deletion mutants used in the RNA pull‐down and RIP assays shown in (g,h). g) Western blot of the indicated Flag‐tagged KPNA2 deletion mutants retrieved by in vitro transcribed tRF‐5004b obtained from 293T cell extracts (*n* = 3). h) qRT‐PCR analysis of endogenous tRF‐5004b enriched by the indicated Flag‐tagged KPNA2 deletion mutants in 293T cells (*n* = 3). U6 mRNA served as the negative control. i) Schematic representation of different KPNA2 mutants used in the RNA pull‐down and RIP assays shown in (j,k). j) Western blot of the indicated Flag‐tagged different KPNA2 mutants retrieved by in vitro transcribed tRF‐5004b obtained from 293T cell extracts (*n* = 3). k) qRT‐PCR analysis of endogenous tRF‐5004b enriched by the indicated Flag‐tagged KPNA2 deletion mutants in 293T cells (*n* = 3). U6 mRNA served as the negative control.

To further decipher the molecular basis for tRF‐5004b binding with KPNA2, we performed a series of computational simulations to analyze KPNA2‐tRF‐5004b complex formation. KPNA2 is composed of 529 amino acids, containing an importin‐β binding domain in the N‐terminus; two NSL binding domains, namely, a minor NSL domain and a major NSL domain in the middle; and a CAS binding domain at the C‐terminus.^[^ ^26^ ^]^ The Gibbs free energy landscape plot revealed the deepest energy well between KPNA2 and tRF‐5004b, indicating that tRF‐5004b may bind to the NSL region of KPNA2 in a manner that represents the most stable binding mode (**Figure** **5**a).

We further performed an RNA pull‐down assay using a series of Flag‐tagged KPNA2 constructs with deletions on the basis of structural features to map its functional motif with tRF‐5004b (Figure 5b). Consistent with the computer simulation results, the deletion of the NSL of KPNA2 strongly impaired the interaction of KPNA2 with tRF‐5004b (Figure 5c). RIP analysis also revealed the high affinity of NSL for tRF‐5004b and a truncated KPNA2 fragment without NSL bound to tRF‐5004b with lower efficiency (Figure 5d). These results suggest that the NSL domain is necessary and sufficient to mediate the interaction between KPNA2 and tRF‐5004b.

The binding sites of KPNA2‐tRF‐5004b were characterized through free energy calculations, identifying the THR277 site as having the highest binding affinity (Figure 5e). According to the binding sites and the structural characteristics of NSL in this binding mode, the NSL region was further divided into three subregions for RNA pull‐down (Figure 5f). The deletion of residues 239–314 within the NSL domain strongly impaired the interaction between KPNA2 and tRF‐5004b, as shown by the RNA pull‐down and RIP assays (Figure 5g,h), suggesting that the binding sites of tRF‐5004b fall into this region. The region of residues 239–314 was further divided into two subregions on the basis of the binding sites for mutant constructs, one for residues 240–244 (Mut1) and the other for residues 277–279 (Mut2) (Figure 5e,i). Three key residues, K277, T278, and N279, mutated to Ala, resulted in the failure of probe‐based KPNA2 capture, suggesting that these residues are responsible for tRF‐5004b binding (Figure 5j,k). Together, these results suggest that tRF‐5004b interacts with the NSL 239–314 aa region of KPNA2 and that K277, T278, and N279 are key residues responsible for binding.

### 2.6 Targeting tRF‐5004b Relieves Immune Cell Recruitment and Inflammation in the Lung

**Figure 6:**
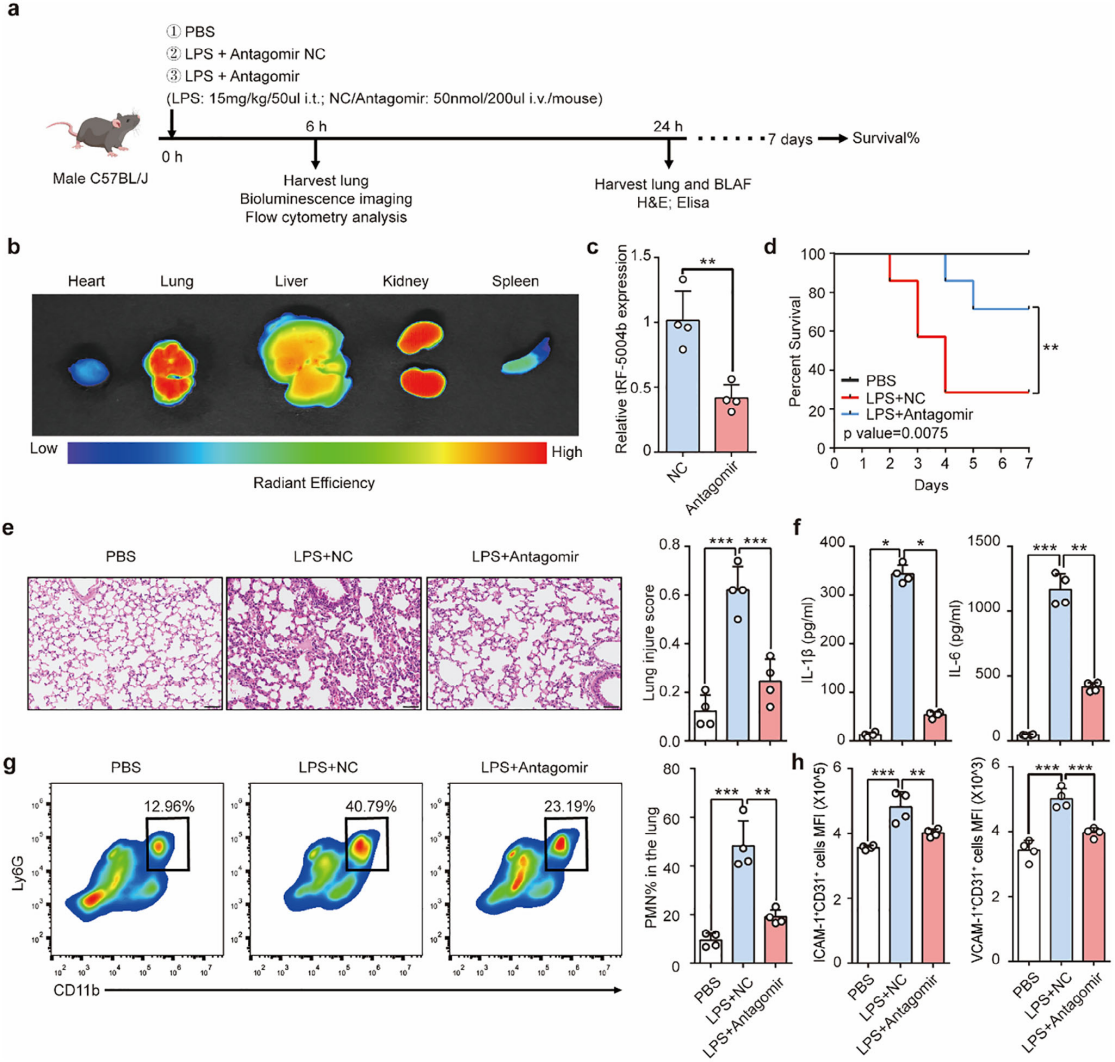
The therapeutic effect of tRF‐5004b antagomir in mouse acute lung injury model. a) Schematic for the treatment of LPS‐induced acute lung injury mice that were treated with PBS, antagomir NC, or antagomir tRF‐5004b. b) Bioluminescence images showing the distribution of antagomir tRF‐5004b in different organs at 6 h after injection. c) qRT‐PCR analysis of tRF‐5004b expression in lung from mice receiving antagomir NC or antagomir (*n* = 3). d) Survival analysis of mice according to treatment (*n* = 7 for each group). e) Representative images of H&E‐stained lung sections in mice and lung injury score at 12 h (*n* = 4). f) IL‐1β and IL‐6 levels in each group of lung tissue were analyzed using ELISA (*n* = 4). g) Flow cytometric analysis of the proportion of PMNs in lung tissue from each group (*n* = 4). h) MFI of ICAM1 and VCAM‐1 in ECs after treatment with antagomir NC or antagomir (*n* = 4). Statistics: unpaired two‐tailed *t*‐test in (c); Log‐rank test in (d); one‐way ANOVA with Tukey's multiple‐comparisons test or Kruskal–Wallis test with Dunn's multiple comparison tests in (e–h). Data are represented as mean ± SEM. *p < 0.05, **p < 0.01 and ***p < 0.001.

Given that tRF‐5004b acts as an inflammatory tsRNA in ARDS, we hypothesized that the inhibition of tRF‐5004b has a therapeutic effect on ARDS. We synthesized a tRF‐5004b‐targeting antagomir with cholesterol modification optimized for in vivo experiments (**Figure** **6**a). As illustrated in Figure 6b,c, intravital imaging revealed the substantial accumulation of the antagomir in the lungs, accompanied by a significant reduction in the expression of lung tRF‐5004b at 6 h post‐injection of the inhibitor via the tail vein. We found that the mice that received the antagomir had a significantly higher 7‐day survival rate and a greater degree of lung injury than did the mice that received the antagomir NC (Figure 6d,e). The levels of inflammatory cytokines (IL‐1β and IL‐6) in alveolar lavage fluid were also significantly reduced in the tRF‐5004b‐inhibitor group (Figure 6f). Moreover, there was a notable decrease in the proportion of lung neutrophils after antagomir administration (Figure 6g), indicating that tRF‐5004b is a potential therapeutic target for ARDS. Consistent with the role of tRF‐5004b in controlling cell adhesion, an analysis of molecules downstream of tRF‐5004b in ARDS revealed that tRF‐5004b antagomir administration decreased the levels of ICAM‐1 and VCAM‐1 in the pulmonary vascular endothelium (Figure 6h). Together, these data support the idea that tRF‐5004b is a previously unrecognized and important target for ARDS lung injury through the inhibition of endothelial adhesion molecules.

### 2.7 tRF‐5004b level in BALF‐SAPs is Significantly Overexpressed in ARDS and Associated with ARDS Progression and Poor Prognosis

**Figure 7:**
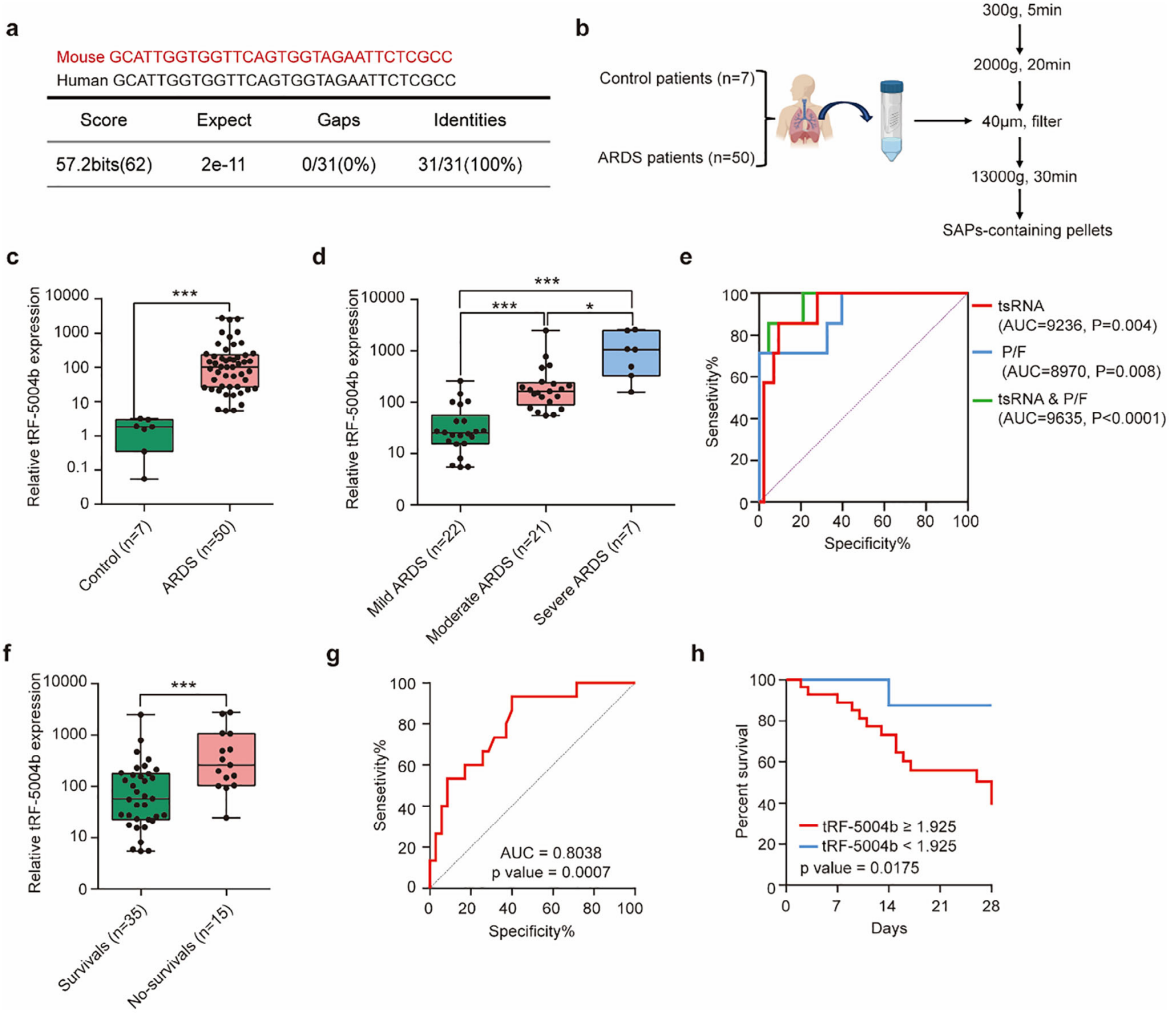
tRF‐5004b levels in BALF‐SAPs correlated with ALI/ARDS severity and prognosis. a) Homology of tRF‐5004b between human and mouse. b) The workflow of BALF‐SAPs isolation. c) The BALF‐SAP tRF‐5004b level in Day 1 ARDS patients (*n *= 50) and controls (*n* = 7). d) The BALF‐SAP tRF‐5004b level detected between patients with different severities of ARDS (mild ARDS, *n* = 21; moderate ARDS, *n* = 22; severe ARDS, *n* = 7). e) The diagnostic value of tsRNA, P/F ratio, and the combination of these metrics in the diagnosis of patients with severe ARDS was analyzed using ROC curves (*n* = 50). f) The BALF‐SAP tRF‐5004b level in survivors (*n* = 35) and non‐survivors (*n* = 15) on Day 1. g) The ability of the BALF‐SAP tRF‐5004b level on Day 1 to predict mortality in patients with ARDS (*n* = 50). h) The survival rate of patients stratified with a cutoff value of 1.925 for BALF‐SAP tRF‐5004b level on Day 1 was analyzed by Kaplan–Meier curves (*n* = 50). Statistics: unpaired two‐tailed *t*‐test or two‐tailed Mann–Whitney *U*‐test in (c, d, f); Multiple regression analysis in (e,g); Log‐rank test in (h). Data are represented as mean ± SEM. *p < 0.05 and ***p < 0.001.

Building upon the aforementioned findings, we proceeded to the clinical setting to validate the alterations in the expression of tRF‐5004b in human samples. Notably, the tRF‐5004b gene in mice orthologous to that in humans (**Figure** **7**a). We aimed to investigate the correlations between tRF‐5004b levels and the severity, prognosis, and degree of lung injury in patients with ARDS. We first isolated BALF‐SAPs from patients with or without ARDS (Figure 7b). The level of tRF‐5004b in the collected BALF‐SAPs was higher in the ARDS group compared to the non‐ARDS group (as a control group) (Figure 7c). Similar findings were identified in an independent cohort of CD68+ MSAPs, which were isolated from BALF‐SAPs through positive selection using magnetic beads (Figure S19a,b, Supporting Information). We then conducted a subgroup analysis according to ARDS severity and found that the level of tRF‐5004b significantly increased with increasing ARDS severity (Figure 7d). The mortality rate among patients with severe ARDS is particularly high,^[^ ^2^ ^]^ underscoring the importance of accurate identification of severe ARDS to improve clinical outcomes. Clinically, the PaO2/FiO2 (P/F) ratio is employed to evaluate ARDS severity. To assess the diagnostic efficacy of tRF‐5004b for identifying patients with severe ARDS, we conducted an ROC curve analysis comparing tRF‐5004b levels against P/F ratios in ARDS patients. Notably, tRF‐5004b levels yielded a higher AUC (0.923, 95% CI 0.838‐1.000, *p* = 0.004) compared to the P/F ratio alone (0.897, 95% CI 0.767–1, *p* = 0.008). Importantly, the combination of these metrics resulted in the highest AUC (0.964, 95% CI 0.903–1.000, *p* < 0.0001) (Figure 7e). Additionally, the tRF‐5004b level was positively correlated with the lung injury score (Figure S20, Supporting Information).

When ARDS survivors compared with non‐survivors, the level of tRF‐5004b was higher in non‐survivors than in survivors (Figure 7f). The univariate and multivariate analyses showed that tRF‐5004b level was an independent risk factor for 28‐day mortality (Tables S5,S6, Supporting Information). Moreover, ROC analysis was performed to test the specificity and sensitivity of tRF‐5004b levels for distinguishing ARDS survivors from non‐survivors. The AUC for tRF‐5004b levels was 0.8038 (95% CI 0.6760–0.9316, *p* = 0.0007, Figure 7g), and the optimal cut‐off value was 1.925. We used the optimal cut‐off value to divide the patients into a high tRF‐5004b group (tRF‐5004b levels ≥ 1.925) and a low tRF‐5004b group (tRF‐5004b levels < 1.925). Kaplan–Meier survival curves showed that the high tRF‐5004b group was at greater risk of death than the low tRF‐5004b group (*p *= 0.0175, Figure 7h). Collectively, these results demonstrate that tRF‐5004b might be a promising biomarker for the diagnostic and prognostic in ARDS patients.

## 3 Discussion

**Figure 8:**
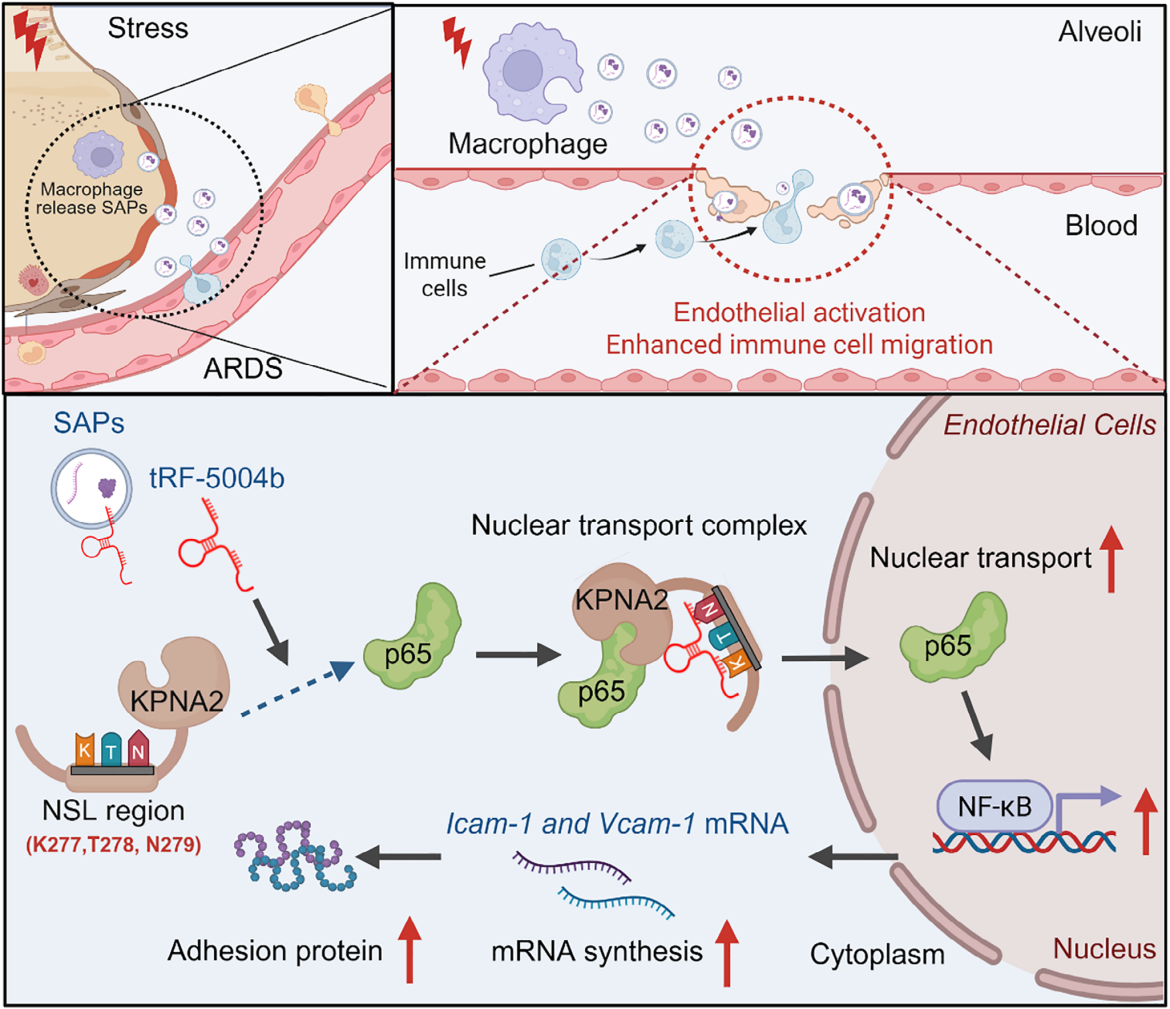
Working model of the role and underlying mechanism of tRF‐5004b‐enriched SAPs in ARDS. The excessive release of tRF‐5004b‐enriched SAPs from inflamed macrophages exacerbates endothelial activation, thereby intensifying inflammation and acute lung injury. Mechanistically, tRF‐5004b facilitates the binding of KPNA2 to p65, thereby enhancing the nuclear translocation of p65. The illustration in Figure 8 was created and licensed using BioRender.com.

EVs, potential key elements in inflammation regulation networks, are reshaping the modern pathophysiology of ARDS.^[^ ^9^, ^27^ ^]^ In this study, we identified an unconventional mechanism through which macrophages regulate the immune function of the pulmonary microvascular endothelium via tRF‐5004b‐enriched SAPs in ARDS. ECs activated by SAPs were observed to rapidly recruit neutrophils to the lung, thereby exacerbating inflammation. Mechanistically, tRF‐5004b was identified as a potential key molecule involved in the pathological process of endothelial activation, as evidenced by transcriptomic profiling and biological validation. tRF‐5004b elicits complex formation with KPNA2 and p65 through RNA–protein interactions, thereby promoting p65 nuclear translocation and accelerating EC activation (**Figure** **8**). Moreover, at the clinical level, we confirmed that an elevated level of tRF‐5004b in SAPs was associated with aggravated ARDS severity and a poor survival rate. Thus, our findings may provide mechanistic insights into how SAPs result in endothelial activation in ARDS and represent an opportunity to open exciting new avenues for improved ARDS therapeutic intervention.

Accumulating evidence has recently indicated that the composition of EVs is not merely the result of a passive or random process of combination and encapsulation; instead, it is cell‐specific and regulated by a series of intricate mechanisms. However, the sorting mechanisms and efficiencies of cargo into EVs and EV‐subpopulations are still poorly understood. Recent studies have shown that some specific short nucleotide sequences, termed “motifs,” are associated with the export of a subset of miRNAs in a particular cell type. Furthermore, selectivity in miRNA loading is mediated by RNA‐binding proteins, which recognize specific motifs of miRNAs. For example, HNRNPA2/B1 can specifically bind to the “GGCU” sequence in miRNA and facilitate its selection into T cell EVs.^[^ ^21b^ ^]^ Similarly, hepatocytes can specifically recognize miRNAs containing the “GCUG” sequence through the RNA‐binding cytoplasmic protein SYNCRIP, thereby promoting their sorting into EVs.^[^ ^21c^ ^]^ Our data indicate that SAPs‐tsRNA expression levels are drastically altered following inflammatory stimulation, during which tRF‐5004b may regulate endothelial cell activation. This finding indicates that a particular sorting mechanism might exist to package tRF‐5004b into SAPs and release it for extracellular activity. LC3II, a membrane surface marker of SAPs, is reported to be essential for the loading and secretion of RNA cargo by binding to RBPs in EVs.^[^ ^23^ ^]^ Interestingly, we identified a molecular pathway wherein LC3II mediates the selective sorting of tRF‐5004b by interacting with HNRNPA2B1, which specifically recognizes the UAG motif within tRF‐5004b. These findings collectively suggest that the selective packaging of tRNAs into EVs is governed by a sophisticated, motif‐driven recognition system involving RNA‐binding proteins and autophagy‐related machinery, opening new avenues for understanding EV‐mediated intercellular communication in ARDS.

Currently, the study of tsRNAs in lung injury remains in a nascent stage. In acute lung injury, it has been reported that exosomes containing tRF‐22‐8BWS7K092, derived from alveolar macrophages, induce ferroptosis in lung epithelial cells.^[^ ^28^ ^]^ Another study revealed that tRF‐Gly‐GCC inhibits cell proliferation and induces apoptosis in cases of radiation‐induced lung injury.^[^ ^29^ ^]^ Additionally, dexmedetomidine has been shown to mitigate lung damage by reducing inflammation, pulmonary oedema, and ferroptosis, leading to alterations in tsRNA expression profiles.^[^ ^30^ ^]^ Changes in tsRNA expression were also observed during mesenchymal stem cell treatment for lung remodeling in COPD mice.^[^ ^31^ ^]^ In this study, we identified tRF‐5004b as a potential therapeutic target for ARDS via the regulation of EC activation, expanding our knowledge of the repertoire by which functional tsRNAs modulate ARDS pathogenesis. The roles and mechanisms of tsRNAs in ARDS still necessitate further investigation.

tsRNAs are implicated in various aspects of biological regulation contingent upon their specific downstream targets. Some tsRNAs perform functions analogous to those of microRNAs (miRNAs), which bind directly to target mRNAs to modulate their stability.^[^ ^32^ ^]^ Apart from regulating mRNA stability, tsRNAs can regulate translation under stress conditions by opening intra‐mRNA secondary structures.^[^ ^13a^ ^]^ Additionally, tsRNAs can also regulate protein function by binding post‐translational modification sites of proteins. For example, tRF‐21 has been shown to decrease DDX17 phosphorylation levels by binding to the phosphorylation site on proteins.^[^ ^33^ ^]^ In this study, we discovered that tRF‐5004b enhances the binding affinity of KPNA2 for the transcription factor p65 by interacting with the functional region of the KPNA2 protein. This interaction facilitates the nuclear localization of p65, thereby activating endothelial function. To the best of our knowledge, this study is the first to demonstrate that tRF‐5004b is a cytosolic factor that facilitates the transport of transcription factors into the nucleus. These findings significantly advance our understanding of the mechanism by which tsRNAs influence protein subcellular localization. Furthermore, KPNA2 is implicated in the regulation of various inflammatory and cancer‐related factors by transporting transcription factors such as P53, STAT3, and E2F into the nucleus.^[^ ^34^ ^]^ Therefore, it is of significant interest to explore whether tRF‐5004b facilitates the nuclear translocation of other key transcription factors via KPNA2 for essential biological functions.

The diagnosis and prognosis of ARDS mainly rely on clinical data and physiological variables with low predictive accuracy. The incorporating of biomarkers into the clinical evaluation of ARDS may deliver better stratification as well as improve the prognostic workup.^[^ ^35^ ^]^ As blood often provides a rapid and convenient window into human immune state evaluations, the levels of inflammatory markers in peripheral blood are frequently utilized to assess the inflammatory status of ARDS patients. However, the compartmentalization of immune responses in ARDS is well appreciated.^[^ ^36^ ^]^ In ventilated patients presenting with lung infiltrates, alveolar cytokine profiles can effectively distinguish between bacterial infections and non‐infectious conditions, whereas circulating cytokine levels are unrevealing. Identifying ARDS subgroups using BALF measurements is a unique approach that complements information obtained from plasma, with the potential to inform enrichment strategies in trials of lung‐targeted therapies.^[^ ^37^ ^]^ In this study, BALF was collected from ARDS patients without additional invasive testing. Elevated tRF‐5004b levels in SAP were associated with the aggravation of ARDS patient degree and poor outcomes. Overall, the use of BALF SAP‐tRF‐5004b as an indicator of the severity and prognosis of ARDS is a powerful complement to the compartmentalization of immune responses in ARDS.

Notwithstanding the advances made here, our study has several limitations. First, this study investigated only the role of tRF‐5004b in ARDS. The potential effects of other tsRNAs on ARDS, as well as the impact of tRF‐5004b on other types of inflammatory diseases, remain uncertain. Furthermore, the clinical studies were limited by small sample sizes, despite our efforts to include a larger cohort of patients with varying severities of ARDS and control subjects. Future research involving a more extensive sample size is necessary to validate and strengthen our findings.

In summary, we elucidated an unreported process of crosstalk between macrophages and ECs mediated by tRF‐5004b‐enriched SAPs and clarified the mechanism by which tRF‐5004b enhances p65 nuclear localization through its interaction with KPNA2, thereby promoting endothelial cell activation and subsequent lung injury. These findings suggest that tRF‐5004b holds remarkable potential as a therapeutic target for ARDS.

## 4 Experimental Section

### Patient Samples Collection

The clinical BALF samples were drawn within 24 h of diagnosis of ARDS. All participants were provided with written informed consent at the time of recruitment. Sample collection was approved by the Ethics Committee of the Zhongda Hospital of Southeast University (2022ZDSYLL411P01). All cases had clinically confirmed diagnoses of ARDS based on the Berlin Definition. The detail baseline characteristics of the patients are summarized in Table S1 (Supporting Information). The BALF samples were stored in −80 °C until used.

### Cell Culture, Transient Transfection, and Establishment of Stable cell Lines

Mouse pulmonary microvascular endothelial cells (iMPMECs) line was constructed in the previous work of the laboratory^[^ ^38^ ^]^ and cultured in DMEM‐F12, containing 5% FBS, 1% PS, 1% ECGS, 100 IU mL^−1^ heparin, and 92 mg L^−1^ D‐valine. Mouse macrophage line RAW264.7, human monocytic cell line THP‐1, human endothelial cell line EAhy926, and human embryonic kidney cell line HEK293T were purchased from the Cell Bank of the Chinese Academy of Sciences (Shanghai, China) and cultured according to standard protocols. All cells were cultured at 37 °C in a 5% CO2 atmosphere. THP‐1 cells were differentiated into macrophage cells through 100 Nm PMA induction for 48 h. Primary bone marrow‐derived macrophages (BMDMs) were obtained from mouse rear legs bone marrow and differentiated to macrophages for 1 week with M‐CSF. Electroporation was used for tsRNA mimics and siRNA transfection, and Lipofectamine 2000 Transfection Reagent (Invitrogen) was used for plasmid transfection. Stable knockdown of tRF‐5004b was established by lentivirus‐mediated expression of the tRF‐5004b antisense sequence, followed by selection for puromycin‐resistant cells. Cells in which tRF‐5004b was overexpressed were generated by electric transfecting tRF‐5004b mimics.

### Isolation, Purification, and Characterization of SAPs

SAPs were isolated and purified according to previous literature.^[^ ^10c^ ^]^ Briefly, after 100 ng mL^−1^ LPS treatment or no treatment, RAW264.7 cells and their cellular debris were removed through centrifugation (2000 rpm, 10 min). The supernatants were collected and centrifuged twice (12 000 × g, 30 min) to harvest crude products containing SAPs. Subsequently, the SAPs‐containing pellets were collected, washed thrice with PBS. Finally, the pellets were resuspended in PBS and stored at −80 °C or used for nanoparticle tracking analysis (NTA), TEM, flow cytometry (FC), or western blotting (WB) analysis. In addition, the supernatant was collected after treating THP‐1‐derived macrophages and BMDMs with or without 1 µg mL^−1^ LPS. SAPs were extracted using a method as described previously and subjected to NTA analysis.

### tsRNA Sequencing

Total RNA was extracted from C‐MSAPs and L‐MSAPs with TRIzol reagent (Invitrogen, USA). To remove RNA modifications that might interfere with the construction of a small RNA library, RNA was ligated with 3′ and 5′‐adapters, and cDNA was synthesized, followed by PCR amplification. The sequencing analysis was performed via the Illumina NextSeq 500 platform (Aksomics, Shanghai, China) according to the manufacturer's protocol. The tsRNA expression levels were measured and normalized to the number of transcripts per million of total aligned tRNA reads (TPM). Paired *P*‐value < 0.05 was considered statistically significant.

### Animal Experiment

Male C57BL/6J mice (7–8 weeks old) were purchased from GemPharmatech (Nanjing, China). All animal studies con‐formed to the National Institutes of Health Guidelines on the Use of Laboratory Animals and were approved by the Institutional Animal Care and Use Committee of the medical school, Southeast University (Approval number: 20220224041). Mice were administered with lipopolysaccharide (15 mg kg^−1^; Sigma, USA) via instilled intratracheal (i.t.) to create an ARDS model. To study the effects of MSAPs, mice were treated with intratracheal instillation of C‐MSAPs/L‐MSAPs (1 × 10^10^ particles mouse^−1^). An equal volume of PBS was used as the negative control. Mice were sacrificed 24 h later to assess lung inflammation and injury. To investigate the uptake of MSAPs by ECs in vivo, mice received an intratracheal instillation of DiD‐labelled SAPs. Lung tissues were taken after perfusion with PBS at 3 h after injection. To detect the role of ICAM‐1 in mediating endothelial cell activation, mice were injected intravenously with either an anti‐ICAM‐1 blocking antibody (14‐0541‐82, Thermo Fisher) or isotype‐matched negative control mAb (14‐4031‐82, Thermo Fisher) at a dose of 2 mg kg^−1^ body weight 2 h before SAPs treatment, and lung tissues were collected 6 h later for further analysis. For tRF‐5004b knockdown in lung tissue, the mice were exposed to a single intravenous injection of 50 nmol antagomir or antagomir NC as a control, and lung tissues and BALF were collected 6 or 24 h later for further analysis. The groups were observed for seven days, and death was recorded every 24 h.

### RNA Sequencing (RNA‑Seq)

The transcriptome sequencing and analysis were conducted by OE Biotech (Shanghai, China). To elucidate the detailed mechanism underlying the activation effect of SAPs on ECs, the ECs were incubated with either C‐MSAPs or L‐MSAPs for 12 h. Furthermore, to further investigate the mechanism of action of tRF‐5004b on ECs, the cells were treated with either SAP‐Vector or SAP‐tRF‐5004b for 12 h respectively. hen, total RNA was extracted from endothelia cells using Trizol reagent (Invitrogen, USA) according to the manufacturer's protocol. RNA purity and quantification were evaluated using a Nanodrop 2000 spectrophotometer (Thermo Scientific, USA). RNA integrity was assessed using the Agilent 2100 Bioanalyzer (Agilent Technologies, USA). Then, RNA‐seq libraries were constructed using the VAHTS Universal V6 RNA‐seq Library Prep Kit and sequenced on an Illumina NovaSeq 6000 platform. The clean reads were mapped to the mouse genome (GRCm38) using HISAT2 (Kim et al., 2015). Transcript abundance was quantified by the fragments per kilobase million. Differential expression analyses were conducted via the DESeq (2012) package for R.

### Co‐Immunoprecipitation

Cells were lysed in IP lysis buffer (87 788, Thermo Fisher Scientific) containing a halt protease inhibitor cocktail (78430, Thermo Fisher Scientific). Cell lysates were incubated with anti‐p65 (6956, Cell Signaling) or anti‐KPNA2 (ab70160, Abcam), antibodies at 4 °C overnight. The immunocomplexes were added to washed Protein A/G magnetic beads (88802, Thermo Fisher Scientific) and incubated at RT (room temperature) for 2 h. The beads were washed five times with IP lysis buffer and subjected to western blot analysis.

### RNA Pulldown and Mass Spectrometry

5′biotin‐labeled RNA probes were synthesized by GenePharma Co. Ltd (Shanghai, China). RNA pull‐down assay was performed using a magnetic RNA protein pull‐down kit (20164, Thermo Scientific) according to the manufacturer's instructions. Briefly, biotin‐labeled tRF‐5004b and control (antisense) probes were mixed with iMPMECs extract (containing 2 mg total protein) in 500 µL RIP buffer and incubated at 4 °C for 2 h. Next, 50 µL of washed streptavidin magnetic beads were added to each reaction and further incubated at RT for another hour. Beads were washed briefly with wash buffer five times and then boiled in SDS loading buffer. Finally, the enriched proteins were separated via SDS‐PAGE and silver stained followed by mass spectrometry (OE Biotech Ltd., Shanghai) and western blotting.

### Protein Expression and Purification

The protein was expressed and purified as previously described.^[^ ^39^ ^]^ Briefly, the mouse KPNA2 PCR product was purified and cloned into pGEM‐T Easy vector (Promega). Then, the resulting vectors were transformed into E. coli (strain BL21). Cells were grown at 37 °C to an Abs600 of 0.6, and KPNA2 expression was induced with 1 mm IPTG at 37 °C for 4 h. Cells were resuspended in 500 mm NaCl, 5 mm imidazole, 20 mm Tris HCl, pH 7.5, supplemented with EDTA‐free protease inhibitor cocktail (Roche) and lysed by sonication. The soluble extract was purified by nickel affinity chromatography, followed by size exclusion chromatography using Superdex 200 columns (GE Healthcare). After concentration, KPNA2 was quantified by absorbance (*ε*280 = 92.71 mM^−1^ cm^−1^), flash frozen and stored at −80 °C.

### In Vitro RNA Pull‐Down

One microgram of recombinant KPNA2 protein was incubated with 50 pmol biotinylated tRF‐5004b RNA probes in RNA‐protein binding buffer for 1 h at RT. Subsequently, the preclear magnetic beads were added to each binding reaction and incubated with rotation at RT for 1 h. Ultimately, the enriched proteins were resolved via SDS‐PAGE and analyzed by western blotting.

### RNA Immunoprecipitation (RIP)

In the RIP experiment, the anti‐FLAG antibody was used along with a BersinBioTM RNA Immunoprecipitation (RIP) Kit (Bes5101) (BersinBio, China) according to the manufacturer's instructions. In Flag‐tagged KPNA2 fragments RIP assays, 1 × 10^6^ 293T cells were transfected into 5 µg indicated plasmid and collected after 48 h. All proteins for RIP were lysed with cell lysis buffer supplemented with Thermo Scientific Halt Protease Inhibitor Cocktail (Thermo Fisher, USA). To prepare antibody‐coated beads, 20 µL Protein A/G magnetic beads were incubated with 1 µg antibody in 500 µL wash buffer at 4 °C for 1 h. Then the beads were washed three times and mixed with the cell lysates in new tubes. The tubes were rotated at 4 °C overnight. Finally, RNA extraction from the beads was further collected by using trizol according to the manufacturer's instructions. Reverse transcription and qPCR were performed as previously described.

### Two‐Step Immunoprecipitation

All processes were performed under RNase‐free conditions. Immunoprecipitation was carried out as described previously. Briefly, ECs with or without Flag‐tagged KPNA2 transfection were treated with L‐MSAPs. ECs subsequently were lysed in a buffer containing 20 mm HEPES (pH 7.8), 400 mm KCl, 5% glycerol, 5 mm EDTA, 1% NP40, protease inhibitors cocktail, and RNase inhibitor. Cell lysates were first immunoprecipitated with an anti‐Flag antibody. Ten percent of the immunoprecipitates were analyzed by western blotting and RT–PCR analysis. The remaining immunoprecipitates were then eluted with Flag peptides (F3290, Sigma–Aldrich). The eluent was further incubated with control IgG or anti‐p65 antibody for a second immunoprecipitation assay, followed by western blot and RT–PCR analysis.

### Lung Intravital Imaging

Two‐photon intravital lung microscopy was performed to visualize PMN recruitment and trafficking as previously described.^[^ ^40^ ^]^ Briefly, Mice were placed on a custom, heated microscope stage. Intubation was performed and general anesthesia was maintained with room air plus 4% isoflurane at a flow rate of 500 mL min^−1^. Thereafter, mice were placed in the right lateral decubitus position and a small incision made to expose the chest cavity. After making a second incision between the fourth and fifth intercostal ribs, the surface of the left lung was exposed for subsequent operation. The 8 mm thoracic window was then inserted into an intercostal incision and a section of the left lung was immobilized against the window with 20–25 mmHg negative pressure. Where indicated, FITC‐dextran (40 mg kg^−1^; FD2000S, Sigma–Aldrich) and PE‐conjugated anti‐mouse Ly6G antibody (25 µg; 551 461, BD) were given intravenous injection before imaging. To visualize the mode and dynamics of PMN migration in the lung, time‐lapse images were taken at 30 s intervals after injection. The accumulated PMNs in observed tissues per field of view were quantified by manual counting at the end of the IVM imaging period.

### Molecular Docking

Protein‐ nucleic acid docking of KPNA2 and tRF‐5004b. First, the KPNA2 protein (PDB ID: 3OQS) was converted to PDB format using AutoDockTools,^[^ ^41^ ^]^ following hydrogenation and charge calculations. Subsequently, protein‐RNA docking was conducted through the HDOCK SERVER online portal.^[^ ^42^ ^]^ HDOCK employed a hybrid algorithm that combined template modeling and ab initio computational free docking for protein‐DNA/RNA docking. The protein PDB files and tsRNA sequences were uploaded to the portal, and docking was executed using the default settings. The protein‐RNA complex models were ultimately generated.

### Molecular Dynamics (MD) Simulations

MD simulation was performed the Amber24 molecular dynamics software package, using the ff19SB force field for the protein, the OL3 force fields for the RNA, and the TIP3P water model. The short‐range electrostatic and van der Waals interactions were truncated at 1 nm, while the long‐range electrostatic interactions were evaluated using the particle mesh Ewald (PME) method. Before production dynamics, the overall model system was energy‐minimized, followed by a 200 ps NVT equilibration and a 100 ps NPT equilibration. Then, the system would run a 100 ns molecular dynamics simulation at a temperature of 300 K and a pressure of 1 bar. All analyses, which included root mean square deviation, root means square fluctuation (RMSF), radius of gyration (Rg), binding free energy were computed using Amber24's Cpptraj module and in‐house Python scripts.

### Luciferase and Reporter Assays

293T (1 × 10^5^) cells were plated in 48‐well plates and transfected with NF‐κB‐luciferase reporter plasmid (firefly luciferase, 50 ng), pRL‐TK plasmid (renilla luciferase, 10 ng) and plasmid encoding MyD88, IKKβ, or p65 together with tRF‐5004b mimics or NC mimics using Lipofectamine 6000 (Beyotime, China). Cells were collected 48 h later and measured with the Dual‐Luciferase Assay (Vazyme, China) with a fluorescence plate reader (PerkinElmer, Inc., Waltham, MA, USA). Data represent relative firefly luciferase activity normalized to renilla luciferase activity.

### Statistical Analysis

Statistical analysis was performed using R version 3.5.3. Differences between the two groups were assessed with a *t*‐test or a two‐tailed Mann–Whitney *U*‐test; when two samples were paired, a paired *t‐*test or a Wilcoxon‐signed rank test were run. Comparisons among multiple groups were performed using one‐way ANOVA with Tukey's multiple‐comparisons test or Kruskal–Wallis test with Dunn's multiple‐comparison test. Pearson's correlation coefficient was used to quantify correlations. Survival was compared using log‐rank analysis. For regression analysis, tRF‐5004b was log‐transformed to improve the model fit, and univariate and multiple regressions were based on the log‐transformed tRF‐5004b levels. For univariate analysis, a list of included variables is available (see Tables S3,S5, Supporting Information). For multiple regression analysis, variables with a *p*‐value less than 0.1 were considered for inclusion in the model selection procedure to determine the final model (see Tables S4,S6, Supporting Information). Stepwise regression was used to arrive at the final model and a *p‐*value of less than 0.05 was taken as statistically significant. All data were presented as the mean of at least three independent replicates. The data were presented as the mean ± SEM (standard error of the mean). Data were considered statistically significant when *p*‐value < 0.05. *p < 0.05, **p < 0.01, and ***p < 0.001 compared to control.

## Conflict of Interest

The authors declare no conflict of interest.

## Author Contributions

X.‐X.Z., H.L., and S.‐F.Z. contributed equally to this work. L.L., W.H., X.‐X.Z., H.L., and S.‐F.Z. conceived the project and designed the experiments. X.‐X.Z. and H.L. conducted the experiments. X.‐X.Z. and S.‐F.Z. contributed to the collection of SAPs. N.L. and K.‐X.L. were responsible for the collection of clinical samples. S.‐K.G., H.‐Q.L., Y.‐T.W., and X.‐Y.X. assisted the animal studies. X.‐X.Z., H.L., and S.‐F.Z. analyzed the results and wrote the manuscript. L.L. and W.H. critically revised and commented on the manuscript. Y.T., L.‐X.W., T.L., J.C., Y.Y., and H.‐B.Q. provided technical support and insightful suggestions. All the authors read and approved the final manuscript.

## Supporting information



Supporting Information

Supplemental Movie 1

Supplemental Movie 2

## Data Availability

The data that support the findings of this study are available from the corresponding author upon reasonable request.
