# Supplementary material for: tRF‐5004b Enriched Secretory Autophagosomes Induce Endothelial Cell Activation to Drive Acute Respiratory Distress Syndrome
Source: Adv Sci (Weinh). 2025 Jun 20;12(33):e03014. doi: 10.1002/advs.202503014 (PMC12412491; doi:10.1002/advs.202503014)
Supplement: Supplementary file 1 — Supporting Information [file ADVS-12-e03014-s001.docx]

Supporting Information

**tRF-5004b Enriched Secretory Autophagosomes Induce Endothelial Cell Activation to Drive Acute Respiratory Distress Syndrome**

**Authors:** *Xing-xing Zhu^1†^, Han Li^1†^, Shuang-feng Zi^1†^, Ying Tang^1^, Na Liu^1^, Ke-xin Li^1^, Shi-ke Geng^1^, Hui-qing Lu^1^, Zhi-kang Xie^1^, Xin-yi Xu^1^, Yi-ting Wei^1,2^, Li-xin Wang^1,2^, Tao Liu^1^, Jie Chao^1,3^, Yi Yang^1^, Hai-bo Qiu^1^, Wei Huang^1*^, Ling Liu^1*^*

**Affiliations:**

1. Jiangsu Provincial Key Laboratory of Critical Care Medicine, Department of Critical Care Medicine, Zhongda Hospital, School of Medicine, Southeast University, Nanjing, Jiangsu, 210009, China.

2. Department of Microbiology and Immunology, School of Medicine, Southeast University, Nanjing, Jiangsu,210009, China.

3. Department of Physiology, School of Medicine, Southeast University, Nanjing, Jiangsu, 210009, China.

**^†^ These authors contributed equally**

***Corresponding authors:**

lingliu@seu.edu.cn to Ling Liu and whuang@seu.edu.cn to Wei Huang, Jiangsu Provincial Key Laboratory of Critical Care Medicine, Department of Critical Care Medicine, Zhongda Hospital, School of Medicine, Southeast University, Nanjing, Jiangsu, 210009, China.

**This file includes:**

Supplementary Methods

Figures S1 to S20

Tables S1 to S10

Movies S1 to S2

**Supplementary methods**

1. **SAPs labelling**

Purified SAPs were labeled with DiD (Invitrogen, USA) in accordance with the manufacturer's protocol. The SAPs were diluted in PBS containing 0.1% BSA and subsequently incubated with DiD at a final concentration of 5 µM for 15 min at 37°C. The resulting mixture was further diluted with PBS and subjected to two rounds of ultracentrifugation at 13 000 × g at 4°C for 30 minutes to eliminate any residual free dye. Finally, the pellet obtained was resuspended in PBS for subsequent SAPs uptake assay in vitro. Purified SAPs were stained by PKH26 staining (Sigma, Sigma Aldrich) in accordance with the manufacturer's protocol. SAPs diluted in 1 mL of diluent C were pipetted onto 4 μL of PKH26 to a final concentration of 4 μM, and incubated for 15 min at 37°C. After incubation, the reaction was stopped by adding an equal volume of complete media and centrifuged at 13 000 for 30 min. The labelled SAPs were used in the SAPs uptake assay in vivo.

**2. Cell adhesion assay**

To measure PMN adhesion, Cytoselect Leukocyte-endothelium adhesion assay kit (Cell Biolabs, San Diego, USA) was utilized according to manufacturer’s instruction. In brief, the iMPMECs were grown to confluence in Gelatin-coated 24-well plates. iMPMECs were pretreated with L-MSAPs for 12 h. The cells were gently washed with serum-free medium, and LeukoTracker™ solution–loaded leukocytes were then added to the ECs. After a 1 h incubation, the endothelial cell monolayer was gently rinsed twice to remove unbound PMNs. The adhesion of PMNs in each group was observed with a fluorescence microscope (Leica, Germany). Then the cells were lysed with lysis buffer. The values of fluorescence intensity were measured with a fluorescence plate reader (PerkinElmer, Inc., Waltham, MA, USA) at 480/520nm and normalized to the control.

**3. Lung histology**

The left lungs were preserved in 4% paraformaldehyde, embedded in paraffin, and sectioned into slices of 5 μm thickness. Following dewaxing and rehydration, the sections underwent hematoxylin-eosin (H&E) staining. Lung injury was assessed in accordance with the international scoring standard [1], which evaluates the following five histological characteristics: (i) infiltration of alveolar neutrophils, (ii) infiltration of interstitial neutrophils, (iii) presence of hyaline membranes, (iv) accumulation of proteinaceous debris within the airspaces, and (v) thickening of the alveolar septa.

**4. wet/dry weight ratio**

As a separate indicator of pulmonary edema formation, the wet-to-dry (W/D) weight ratios were calculated for both the left and right lungs. Each lung was excised individually, and the wet weight was promptly measured. This initial weight was then divided by the corresponding weight obtained after drying the lung tissue for 24 h in an oven set at 65°C.

**5.** **Preparation of lung single cell suspensions**

The half lung was dissected into pieces  and subjected to enzymatic digestion in 2.5 mL of RPMI 1640 medium (Gibco, USA) supplemented with DNase I (50 μg/mL; 10104159001; Roche) and collagenase IV (1 mg/mL; C5138; Sigma-Aldrich).  This process was conducted at 37℃ or 45 minutes using a shaker incubator set at 125 rpm.

Subsequently, the digested tissue was sieved through a 70 μm cell strainer (BD, USA).

Then, the red blood cells were removed using RBC lysis buffer (555899; BD) at 4℃ for 5 min and centrifuged at 400 × g for 5 min. The remaining cells were washed and resuspended in PBS to prepare lung single cell suspensions for flow cytometric staining, analysis and sorting.

**6. PMN isolation from mouse bone marrow**

PMNs were isolated from mouse bone marrow following established protocols[2]. Specifically, PMNs were extracted from the bone marrow of C57BL/6 mice via negative selection, employing the PMN isolation kit (130-097-658, Miltenyi) in accordance with the manufacturer's guidelines. The purity of the isolated PMNs, characterized by the markers CD11b+ and Ly6G+, was verified to exceed 95% through flow cytometry analysis using the LSRFortessa system (BD Biosciences).

**7. RNA preparation and qRT–PCR**

The same amount of Caenorhabditis elegans cel-39-3p mRNA (RiBoBio, China) was spiked into each SAPs sample as an external calibration for RNA extraction, reverse transcription, and tsRNA amplification. Total RNA was extracted from SAPs and cell samples using an Invitrogen™ TRIZOL according to the manufacturer’s instructions. All RNA samples were stored at –80 °C before reverse transcription and quantitative RT-PCR. SAPs RNA was reverse transcribed into cDNA using PrimeScript^TM^ RT regent Kit (Takara, Japan). Cellular RNA was reverse transcribed into cDNA using reverse transcriptase kit (Vazyme). Quantitative RT-PCR was performed using the HiScript II Q RT SuperMix for qPCR (+gDNA wiper) (Vazyme, China) according to the manufacturer’s instructions. Data were normalized to levels of U6 (nuclear RNA), cel-miR-39-3p (SAPs), GAPDH (cytoplasmic RNA) or ACTIN (total cell RNA). The relative expression level for tRF-5004b and other tsRNA or mRNA was determined using the 2−ΔΔCt method. The primers are listed in Supplementary Table S3.

**8. Western blot**

Total protein and nuclear protein were extracted from the prefrontal cortex and hippocampus using the Total Protein Extraction Kit (P1250; Applygen Technologies Inc., Beijing, China) and Nuclear and Cytosol Fractionation Kit (P1200; Applygen Technologies Inc.), respectively, in accordance with the manufacturer's instructions. A BCA protein Assay kit was used to determine protein concentration. Samples of protein lysate were resolved by an 4% to 20% sodium dodecyl sulfate–polyacrylamide gel electrophoresis (SDS-PAGE) gel and electro-transferred to PVDF membranes. Membranes were blocked with 5% fat-free milk for 1 h at RT and probed with a specific primary antibody overnight at 4 °C, followed by secondary antibody for 1 h at RT.

**9. Flow cytometry analysis and cell sorting**

To clarify the uptake of SAPs in the pulmonary endothelium, the lung single cell suspensions were first stained with viability dye (564406, BD) and then incubated with Fc Block (553141, BD) to reduce nonspecific binding, followed by staining with various fluorochrome-labelled antibodies: anti-CD45 (557659, BD) and anti-CD31 (557396, BD). The uptake of SAPs was quantified as the mean fluorescence intensity (MFI) of DiD. To investigate the effects of SAPs on pulmonary endothelium activation, lung single cell suspensions were stained with the following fluorochrome-labelled antibodies after blocking with Fc Block: anti-CD45, anti-CD31, anti-CD54 (155305, BioLegend). Lung single-cell suspensions were prepared as described in the Supplementary. To assess the effect of endothelial activation on recruitment of neutrophils by SAPs, cells were incubated with Fc Block and then stained with fluorochrome-conjugated antibodies against CD45, CD11b and Ly6G (141706, BioLegend). CD31+ endothelial cells, CD326+ epithelial cells, F4/80 macrophage and Ly6G+ neutrophils were sorted from lung single-cell suspensions using a FACSAria cell sorter (BD Bioscience), and the purity of obtained cells was proved using Cytek Aurora spectral flow cytometer. Data were collected using the Cytek Aurora spectral flow cytometer (Cytek, USA) and analysed with FlowJo V10 software (Tree Star, Inc., Ashland, OR, USA).

**10. Confocal Raman Microscopy**

The epithelial cell, endothelial cell, neutrophil, and macrophage images were generated by linear combination modeling using reference spectra from the pure components, as previously described in[3], utilizing a 1 mg ml⁻¹ albumin unit spectrum. All Raman spectra were acquired using a SuperVision Medicine Bio-SV Raman confocal micro-spectrometer equipped with a green solid-state excitation laser (λ = 532 nm), a 50× air objective lens (Zeiss EC Epiplan-Neofluar Dic 50x, numerical aperture=0.55) and a thermoelectrically cooled charge-coupled detector. The laser power was set at 50 mW for all measurements, with an integration time of 0.5 s per spectrum and an xy-spatial resolution of 2um. Raman wavenumber ranges spanning 0–4000 cm⁻¹were recorded. This was achieved through the development of the Raman spectral unmixing technique which applied the non-negative least squares regression to differentiate between different cell types within the tissue specimen.

**11. Immunofluorescence**

For immunofluorescence staining of lung tissues, the lung tissues were cut into 5 μm thick slices and subsequently treated with 0.3% Triton X-100 for 15 min. After blocking with 10% normal goat serum (ZLI-9056, ZSGB-BIO, China) in 0.3% Triton X-100 at room temperature for 1 h, the sections were incubated with Antii-CD31antibodies (ab28362, Abcam) overnight at 4℃. The sections were then incubated with Alexa Fluor 488 donkey anti-rabbit IgG (H+L) (711-545-152, Jackson Immuno Research) for 1 h at room temperature and mounted with DAPI-containing mounting medium (Southern Biotech, USA).

Cellular immunofluorescence was performed to assess the nuclear translocation of p65 in ECs. ECs were seeded in glass-bottom culture dishes and incubated with MSAPs for 3 h. After treatment, the cells were fixed with 4% PFA. Then the cells were permeabilized with 0.3% Triton X-100, blocked with 10% goat serum, and incubated with a primary antibody against p65 (#8242, CST) overnight at 4℃. Then, the cells were incubated with Alexa Fluor 555-conjugated goat anti-rabbit IgG (H+L) (A32732, Invitrogen) at RT for 1 h and mounted with a DAPI-containing mounting medium. Images were captured by a STELLARIS Confocal Microscope (Leica, Germany).

**12.** **ELISA**

Protein levels of IL-1β and IL-6 cytokines in the BLAF were quantified using commercially available ELISA kits (Elabscience, China) following the manufacturer’s instructions. The following ELISA kits were used: mouse IL-1β (E-EL-M0037) and IL-6 (E-EL-M0044).

**13. MSAPs isolation from BALF-SAPs**

After the BALF-SAPs were incubated with anti-human CD68-PE antibodies, MSAPs were screened out with anti-PE magnetic beads and ran through a separation column (Miltenyi Biotec, German). The purity of the isolated MSAPs, characterized by the markers CD68+, was verified to exceed 80% through flow cytometry analysis using the Cytek Aurora spectral flow cytometer (Cytek, USA).

**Reference:**

[1] G. Matute-Bello, G. Downey, B. B. Moore, S. D. Groshong, M. A. Matthay, A. S. Slutsky, W. M. Kuebler, *Am J Respir Cell Mol Biol* **2011**, *44* (5), 725, <https://doi.org/10.1165/rcmb.2009-0210ST>.

[2] Y. Wang, S. Sano, K. Oshima, M. Sano, Y. Watanabe, Y. Katanasaka, Y. Yura, C. Jung, A. Anzai, F. K. Swirski, N. Gokce, K. Walsh, *Circulation* **2019**, *140* (6), 487, <https://doi.org/10.1161/circulationaha.118.038820>.

[3] V. LaLone, A. Aizenshtadt, J. Goertz, F. S. Skottvoll, M. B. Mota, J. You, X. Zhao, H. E. Berg, J. Stokowiec, M. Yu, A. Schwendeman, H. Scholz, S. R. Wilson, S. Krauss, M. M. Stevens, *Cell Rep Methods* **2023**, *3* (4), 100440, <https://doi.org/10.1016/j.crmeth.2023.100440>.


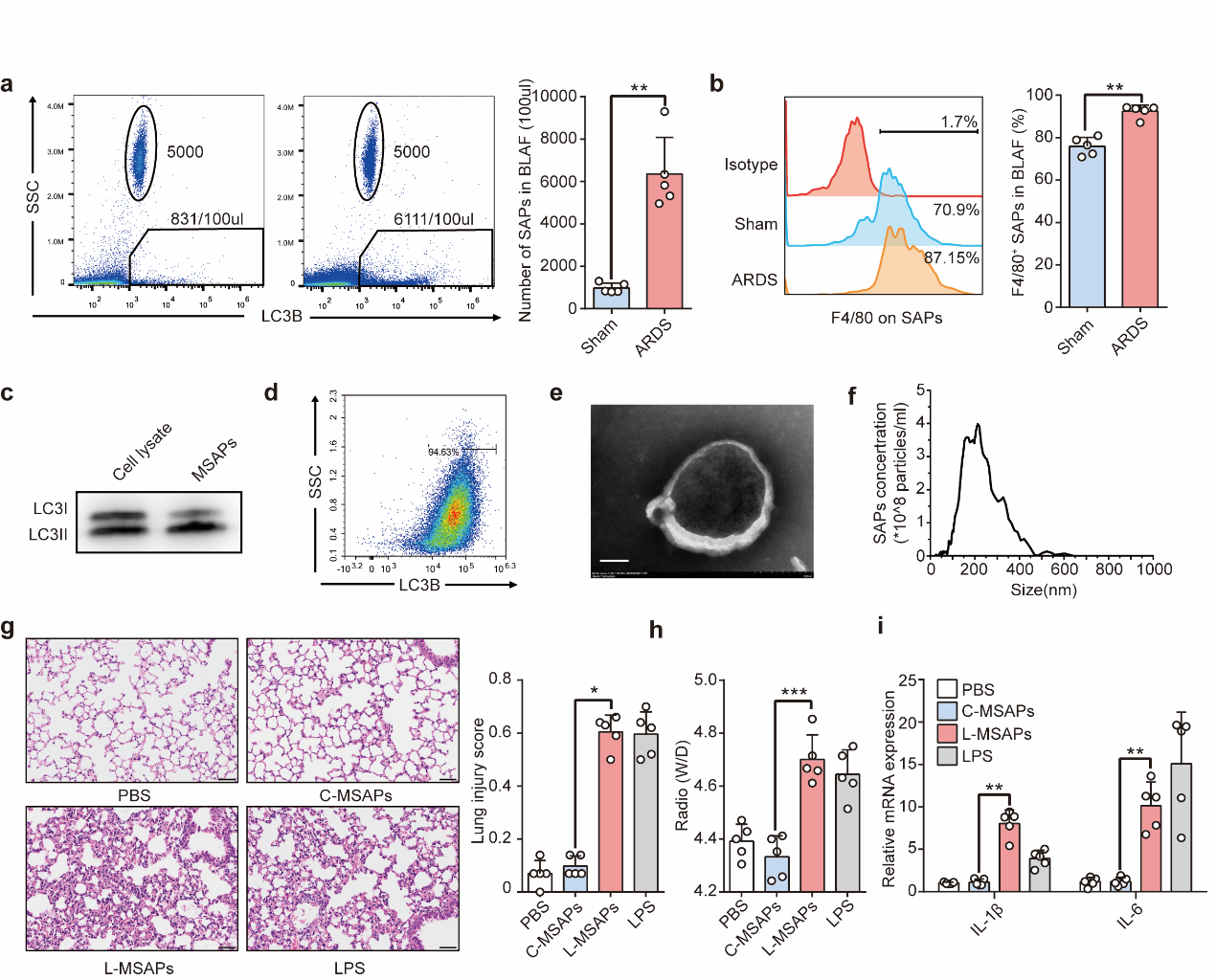
**Figure S1.** L-MSAPs mediate ALI. a) Spectral flow cytometry analysis showing quantification of SAPs isolated from the BLAF of sham and ARDS mice (n=5). b) The percentage of MSAPs (F4/80 positive) in BLAF SAPs (n=5). c,d) The expression of MSAPs marker (LC3) was analyzed by western blotting (c) and flow cytometry (d). e) The morphology of MSAPs was observed by TEM (scale bar: 100 nm). f) The size distribution of MSAPs was analyzed by NTA. g) Mice were injected intratracheally with PBS/C-MSAPs/L-MSAPs/LPS for 24 h, and representative histological images of H&E-stained lung sections are shown (scale bar: 50 μm). The total lung injury score obtained from five independent histological indexes (n=5). h) The wet/dry weight ratio of lung tissue. i) Relative enrichment of proinflammatory cytokines in the lung measured by qRT-PCR (n=5). Statistics: unpaired two-tailed t-test or two-tailed Mann–Whitney U-test in (a, b, g-i). Data are represented as mean ± SEM. **p* < 0.05, ***p* < 0.01, ****p* < 0.001.

**
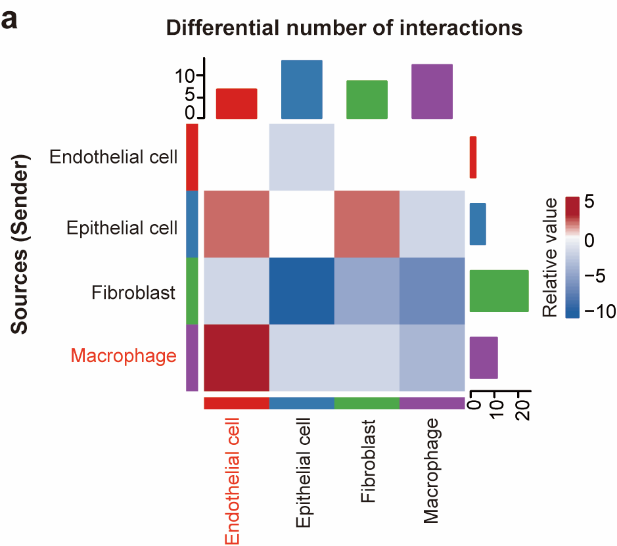
**

**Figure S2.** Analysis of scRNA-seq unveils enhanced crosstalk between macrophages and ECs in acute lung injury. a) CellChat analysis showing intercellular communication between macrophage and lung parenchyma cells.


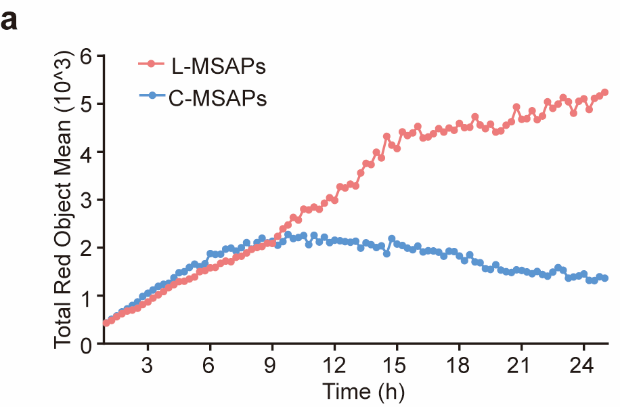


**Figure S3.** L-MSAPs induce endothelia cell activation. a) Quantitative analysis of the amount of MSAPs taken upby endothelia cell over time (n=1).

**
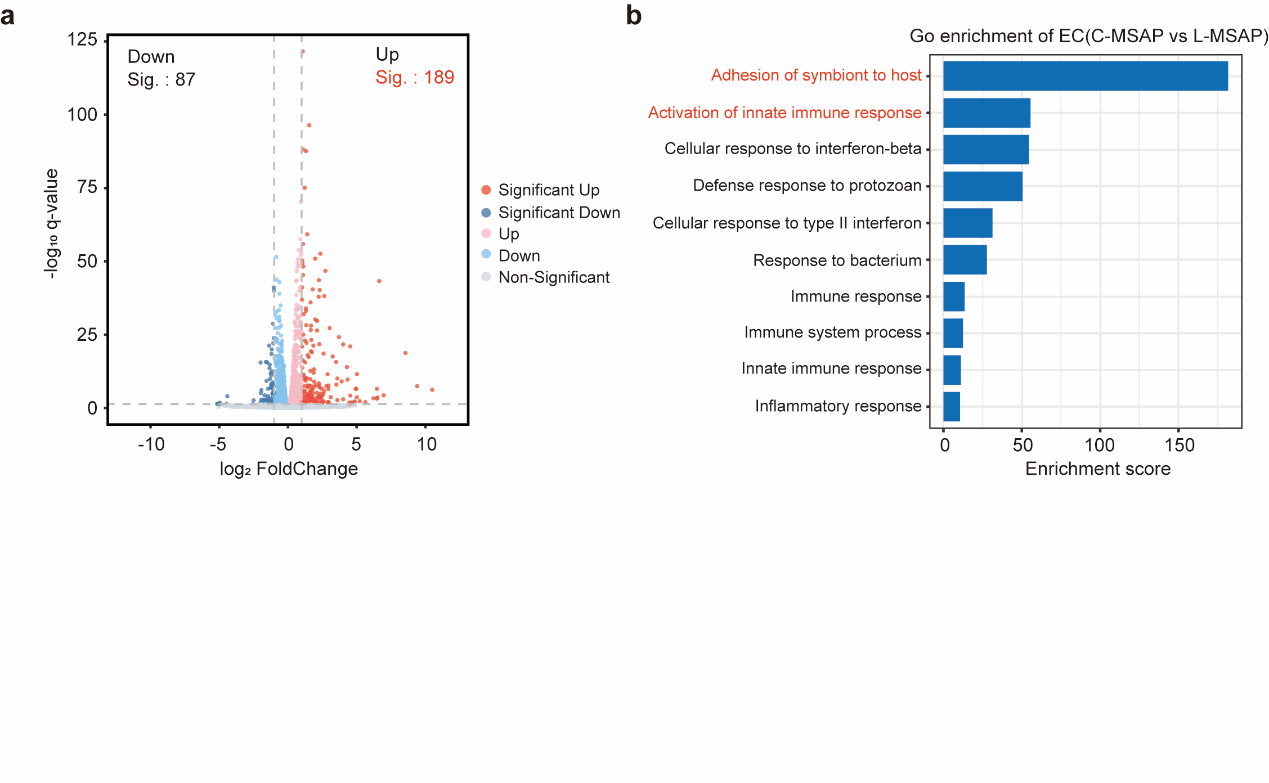
Figure S4.** Transcriptome sequencing data showed that L-MSAPs  primarily regulated the adhesion function of ECs. a) The volcano map for the distribution of differentially expressed genes. Statistical significance was defined as a p value < 0.05 and |log2FC| > 1. b) GO analysis of upregulated diﬀerently expressed genes in ECs treated with L-MSAPs versus the C-MSAPs group.


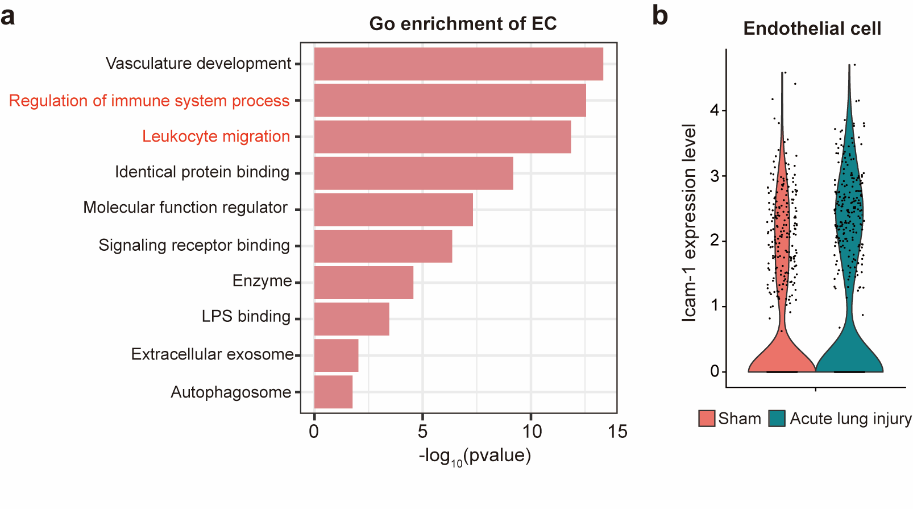


**Figure S5.** RNA-seq analysis revealed that ECs, characterized by the upregulation of adhesion molecules, primarily regulates leukocyte migration in acute lung injury. a) GO analysis of diﬀerently expressed genes in ECs from the lung of the acute lung injury group versus the sham group. b) Violin plots showing expression of *Icam-1* gene in ECs.


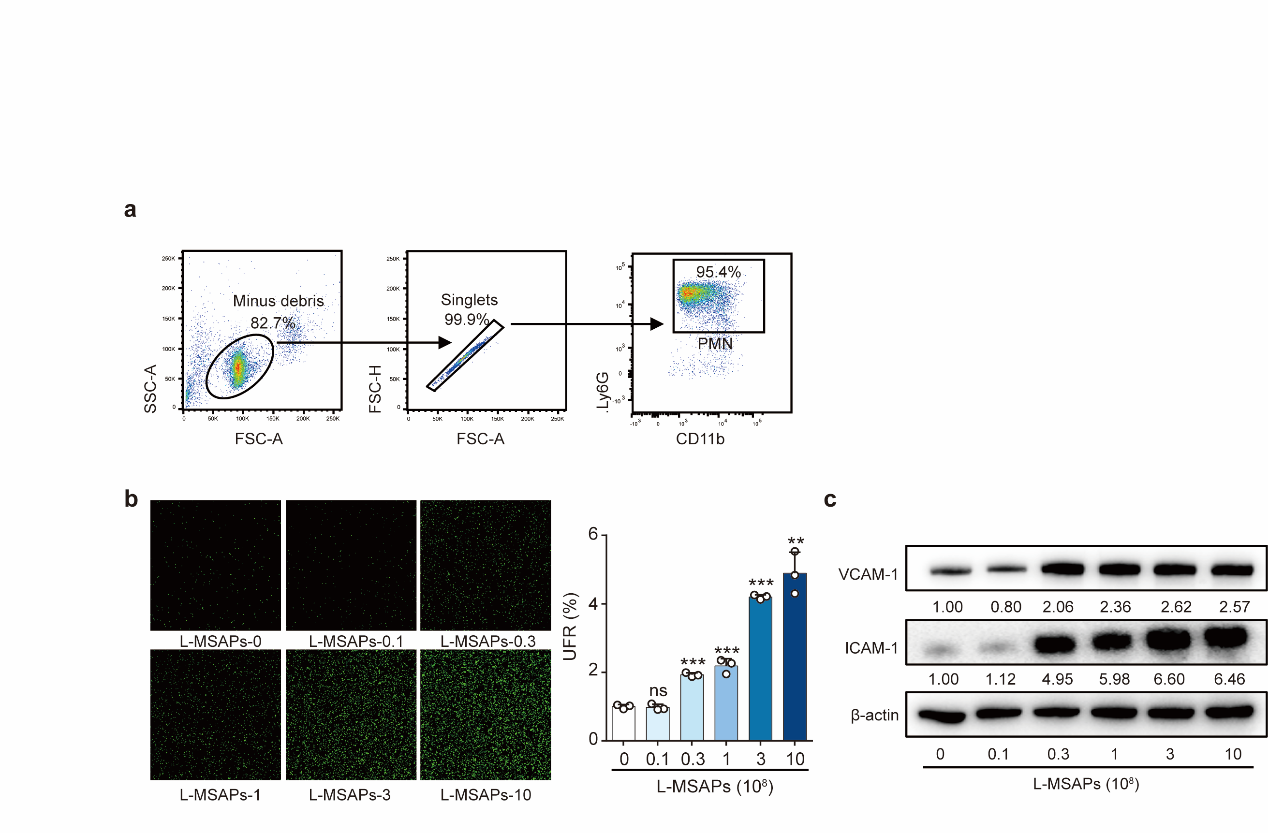
**Figure S6.** L-MSAPs induce endothelial cell activation in a dose-dependent manner. a) Flow cytometry dot maps of purified neutrophil (PMN). b) Representative image of endothelial adhesion PMNs and fluorescence intensity after 0, 0.1, 0.3, 1, 3, 10*10^8 particles/ml L-MSAPs treatment for 12 hours (n = 3). c) Western blotting analysis of ICAM-1 and VCAM-1 in ECs stimulated by 0, 0.1, 0.3, 1, 3, 10*10^8 particles/ml L-MSAPs for 12 h (n=1). Statistics: unpaired two-tailed t-test or two-tailed Mann–Whitney U-test in (b). Data are represented as mean ± SEM. ns, no signiﬁcance. ***p* < 0.01, ****p* < 0.001.

**
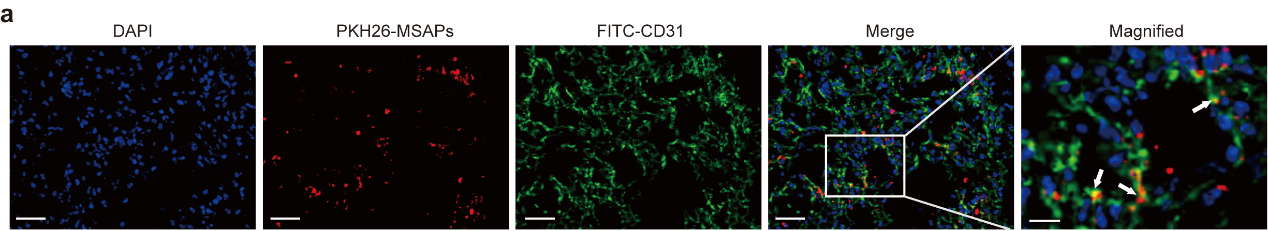
**

**Figure S7.** The uptake of MSAPs by ECs in vivo. a) Colocalization of PKH26-labeled MSAPs (red) and CD31 (green) after intratracheal injection for 3 h. Scale bars: 20 μm (wide ﬁeld) and 5 μm (magniﬁed spot)

**
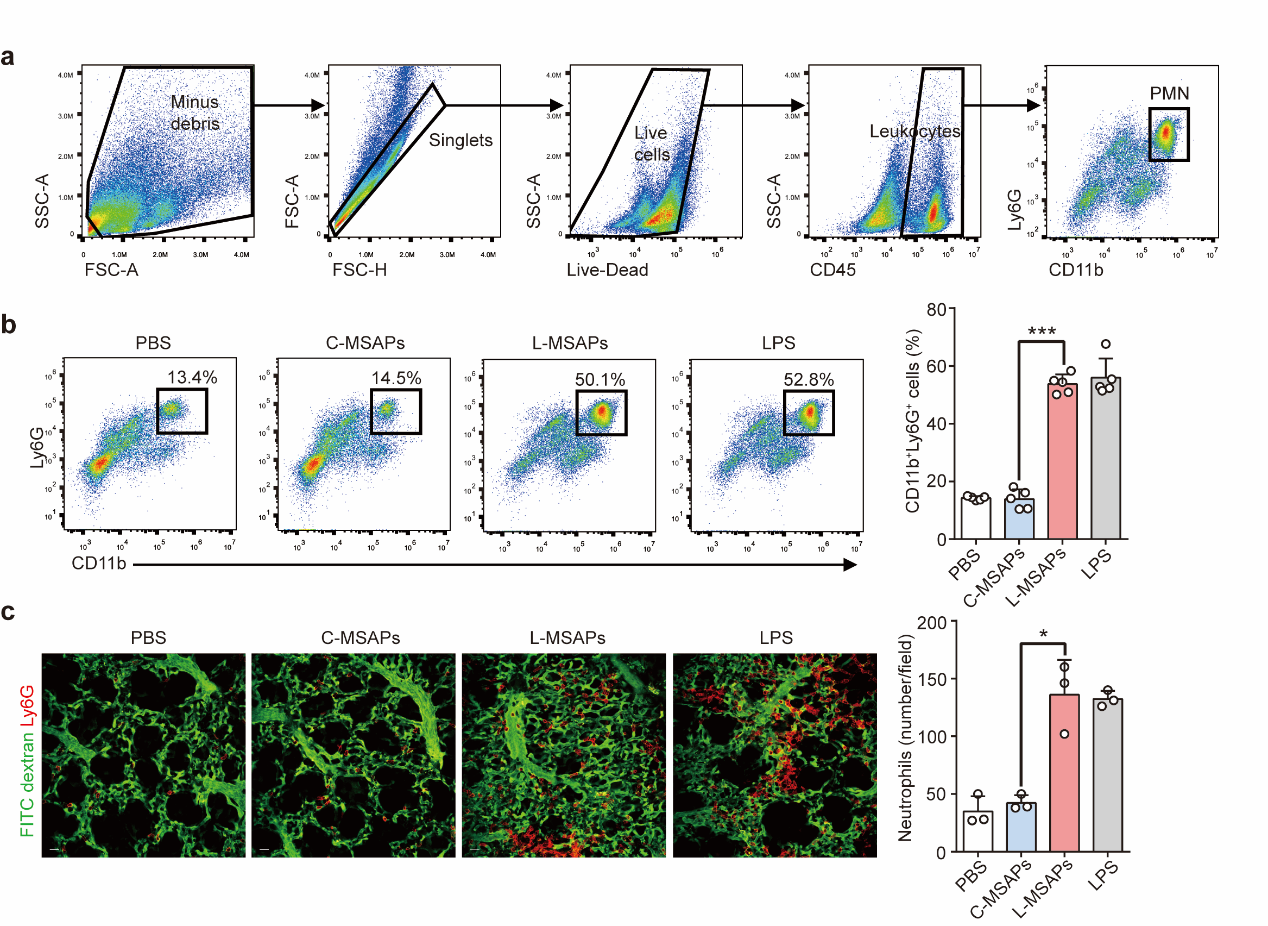
Figure S8.** L-MSAPs mediate endothelia cell to recruit more neutrophils. a) Flow cytometry dot plots of PMN in single cell suspensions of lung tissues are shown. b) Flow cytometry analysis of PMN proportions on the lungs in mice treated with PBS/C-MSAPs/L-MSAPs/LPS (n = 5). c) Representative intravital imaging of pulmonary microcirculation (FITC dextran, green) and PE-labeled Ly6G (red) in mice treated with treated with PBS/C-MSAPs/L-MSAPs/LPS (scale bar: 50 μm). Quantification of PMN infiltration in lungs 6 h after treatment (n = 3). Statistics: unpaired two-tailed t-test or two-tailed Mann–Whitney U-test in (b, c). Data are represented as mean ± SEM. **p* < 0.05, ****p* < 0.001.

**
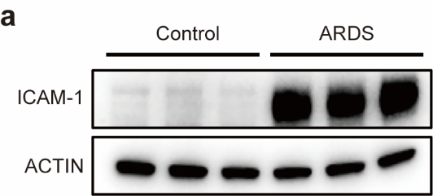
**

**Figure S9.** The effect of BALF-SAPs on adhesion of human vascular endothelial cells. a) Western blotting analysis of ICAM-1 in human vascular endothelial cells treated with BALF-SAPs from control and ARDS patients (n=3).

**
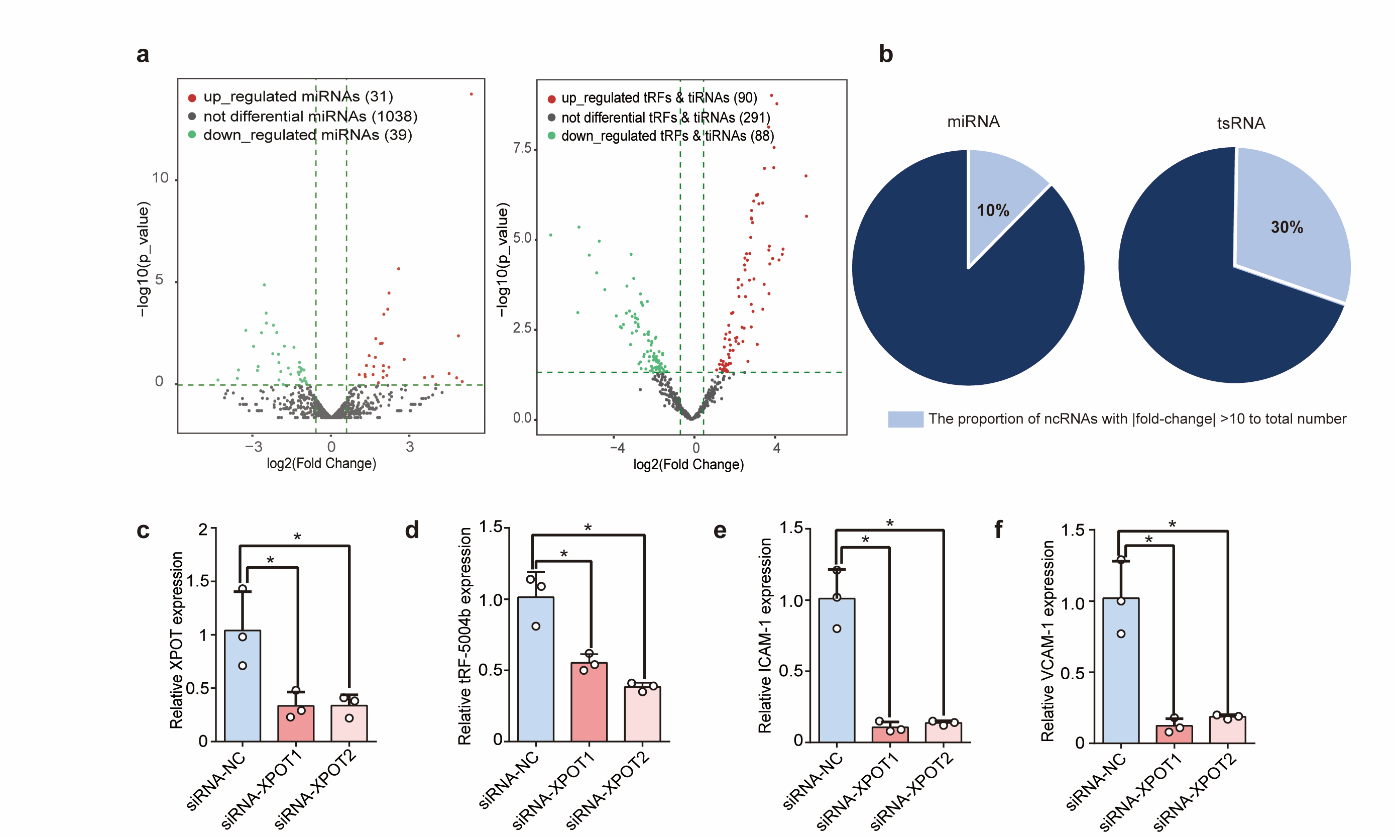
Figure S10.** tsRNA of L-MSAPs contribute to endothelia cell activation. a) Small RNA sequencing (RNA-seq) was employed to identify miRNA and tsRNA expression between C-MSAPs and L-MSAPs groups. b) The proportion of differential miRNA and tsRNA expression( (>10-fold)). c) XPOT-knockdown efficiency of RAW264.7 cells at 24 h (n = 3). d) tsRNA expression of L-MSAPs obtained from XPOT-knockdown RAW264.7 cells or the NC-treated cells stimulated by LPS for 24 h (n=3). e, f) RNA expression of Icam-1and Vcam-1 in the ECs treated with L-MSAPs. Statistics: unpaired two-tailed t-test or two-tailed Mann–Whitney U-test in (c-f). Data are represented as mean ± SEM. ns, no signiﬁcance. **p* < 0.05.


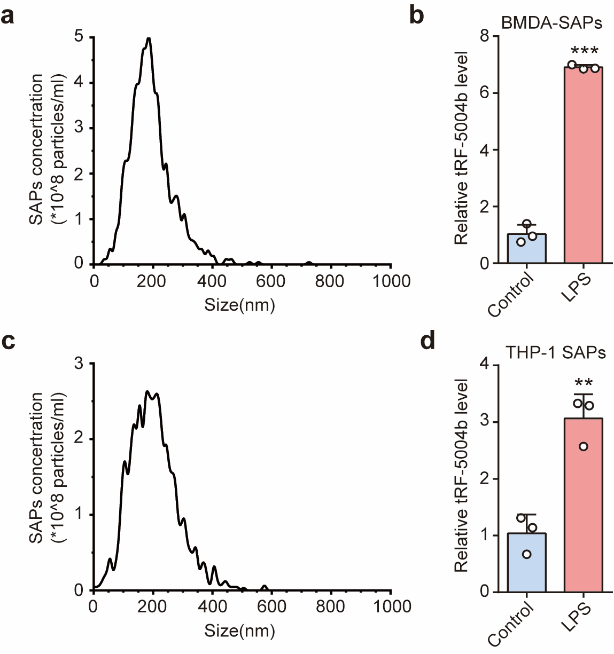


**Figure S11.** The level of tRF-5004b in SAPs secreted by macrophages between control and LPS group. a) The size distribution of SAPs from THP-1-derived macrophages was analyzed by NTA. b) qRT-PCR analysis of tRF-5004b level in SAPs from THP-1-derived macrophages (n=3). c) The size distribution of MSAPs from BMDMs was analyzed by NTA. d) qRT-PCR analysis of tRF-5004b level in SAPs from BMDMs (n=3). Statistics: unpaired two-tailed t-test in (b, d). Data are represented as mean ± SEM. ns, no signiﬁcance. ***p* < 0.01, ****p* < 0.001.


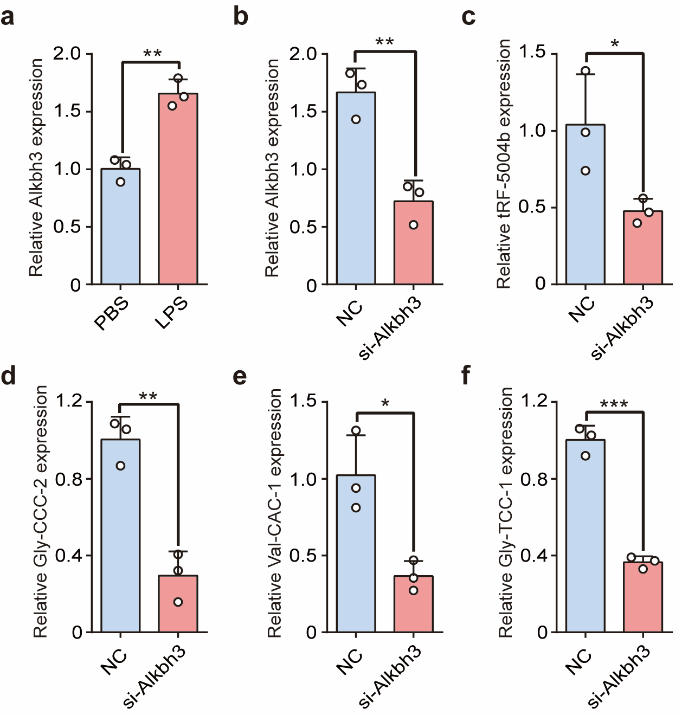


**Figure S12.** The tRNA demethylase ALKBH3 plays a role in the biogenesis of tsRNA within macrophages under conditions of stress. a) Alkbh3 expression in RAW264.7 cells treated with PBS or LPS for 24 h (n = 3). b**)** Alkbh3-knockdown efficiency of RAW264.7 cells at 24 h (n = 3). c-f) The expression of tRF-5004b (c), Gly-CCC-2 (d), Val-CAC-1 (e) and Gly-TCC-1 (f) in Alkbh3-knockdown RAW264.7 cells and NC-treated cells stimulated by LPS for 24 h was measured (n=3). Statistics: unpaired two-tailed t-test or two-tailed Mann–Whitney U-test in (a-f). Data are represented as mean ± SEM. **p* < 0.05, ***p* < 0.01, ****p* < 0.001.


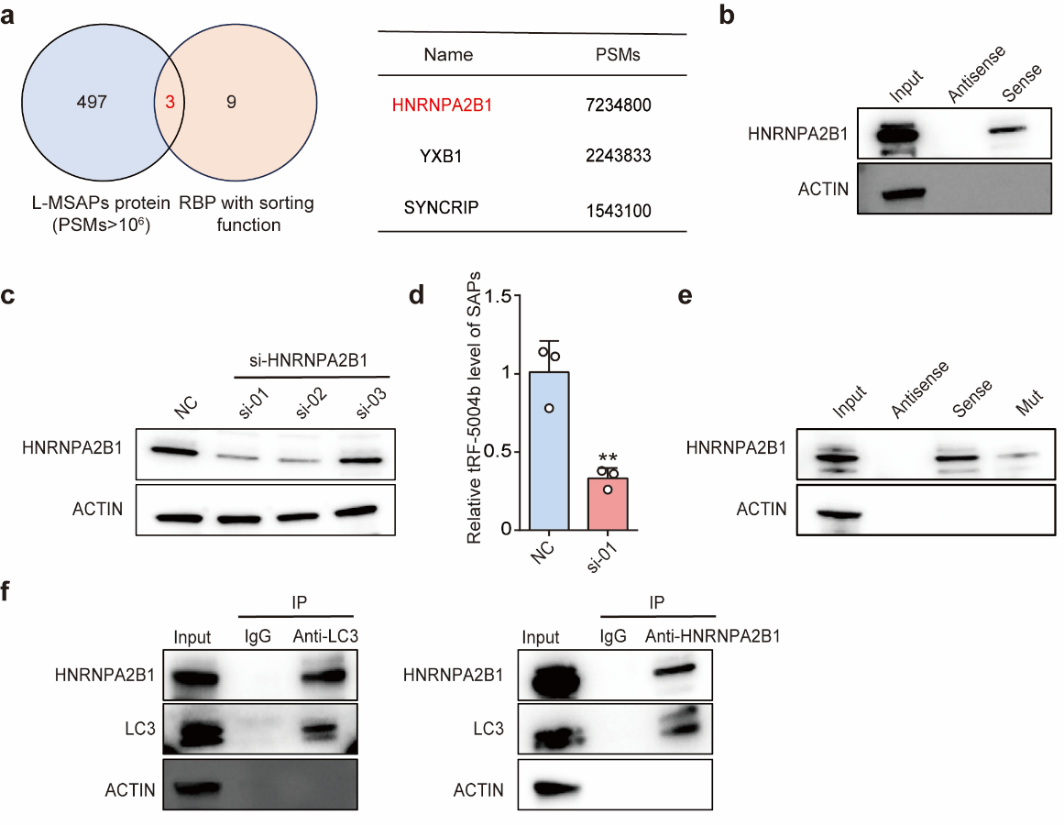


**Figure S13.** HNRNPA2B1 and LC3 play a crucial role in the sorting of tRF-5004b into MSAPs. a) The Venn diagram shows proteins with a numbers of peptide spectrum matches (PSMs) more than 10^6 in L-MSAPs and a subset of RBPs with selecting function. b) HNRNPA2B1 was pulled down by tRF-5004b but not antisense in ECs, as determined by western blotting (n=1). c) HNRNPA2B1-knockdown efficiency of RAW264.7 cells at 24 h (n=1). d) qRT-PCR results of tRF-5004b in MSAPs from RAW264.7 cells transfected with si-01 targeting HNRNPA2B1 for 48 h (n=3). e) HNRNPA2B1 was pulled down by tRF-5004b probe but not Mut probe in ECs, as determined by western blotting (n=3). f) The binding between HNRNPA2B1 and LC3 in RAW264.7 cells (n=1). Statistics: unpaired two-tailed t-test in (d). Data are represented as mean ± SEM. ***p* < 0.01.

**
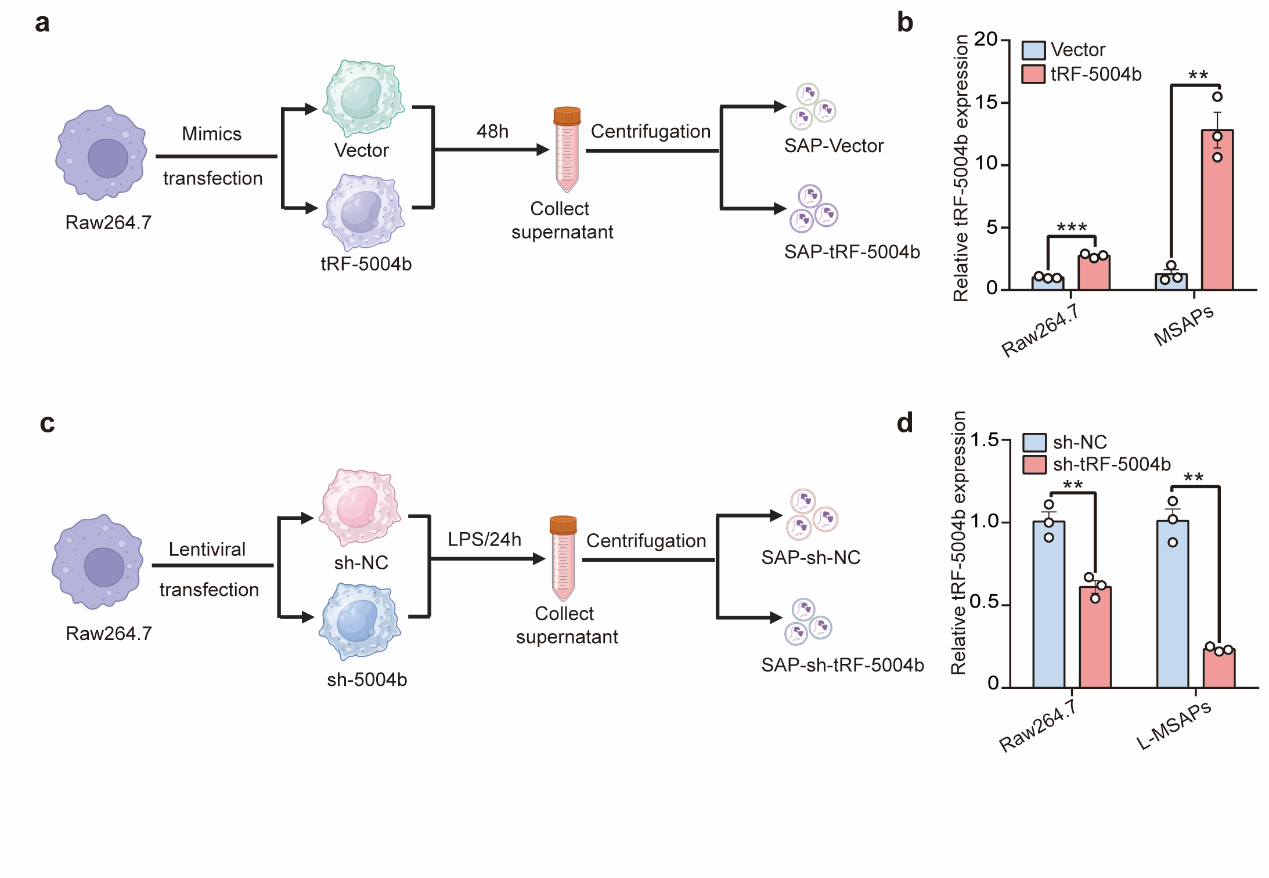
**

**Figure S14.** Construction and verification of tRF-5004b overexpression or knockdown in MSAPs. a) Flowchart for construction of tRF-5004b overexpression in MSAPs. b) The qPCR was used to verify the construction of tRF-5004b overexpression cell and MSAPs models. c) Flowchart for construction of tRF-5004b knockdown in MSAPs (n=3). d) The qPCR was used to verify the construction of tRF-5004b knockdown cell and L-MSAPs models (n=3). Statistics: unpaired two-tailed t-test or two-tailed Mann–Whitney U-test in (b, d). Data are represented as mean ± SEM. ***p* < 0.01, ****p* < 0.001.


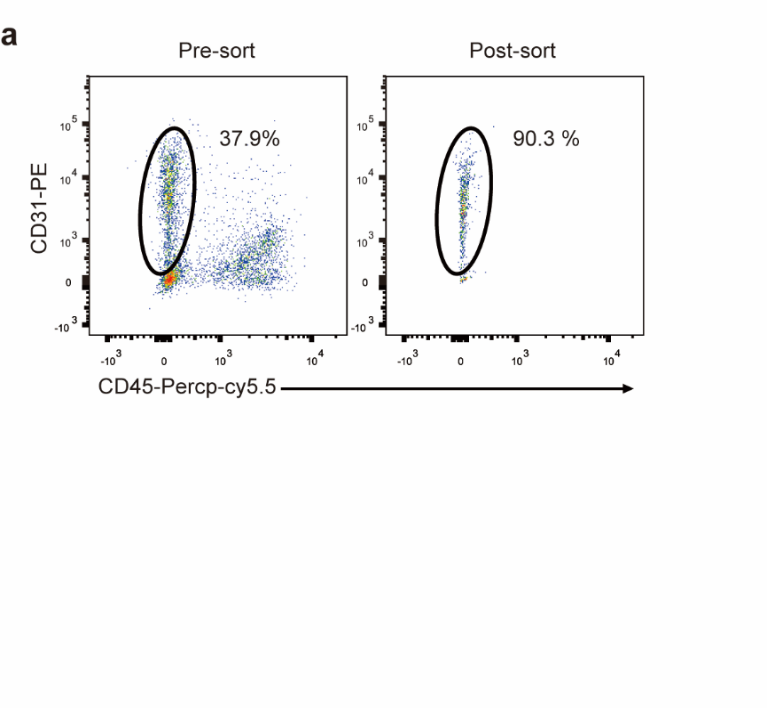


**Figure S15.** ECs purity after sorting was more than 90%. a) Sorted ECs (Live/CD45-/CD31+) were checked for purity by flow cytometry.


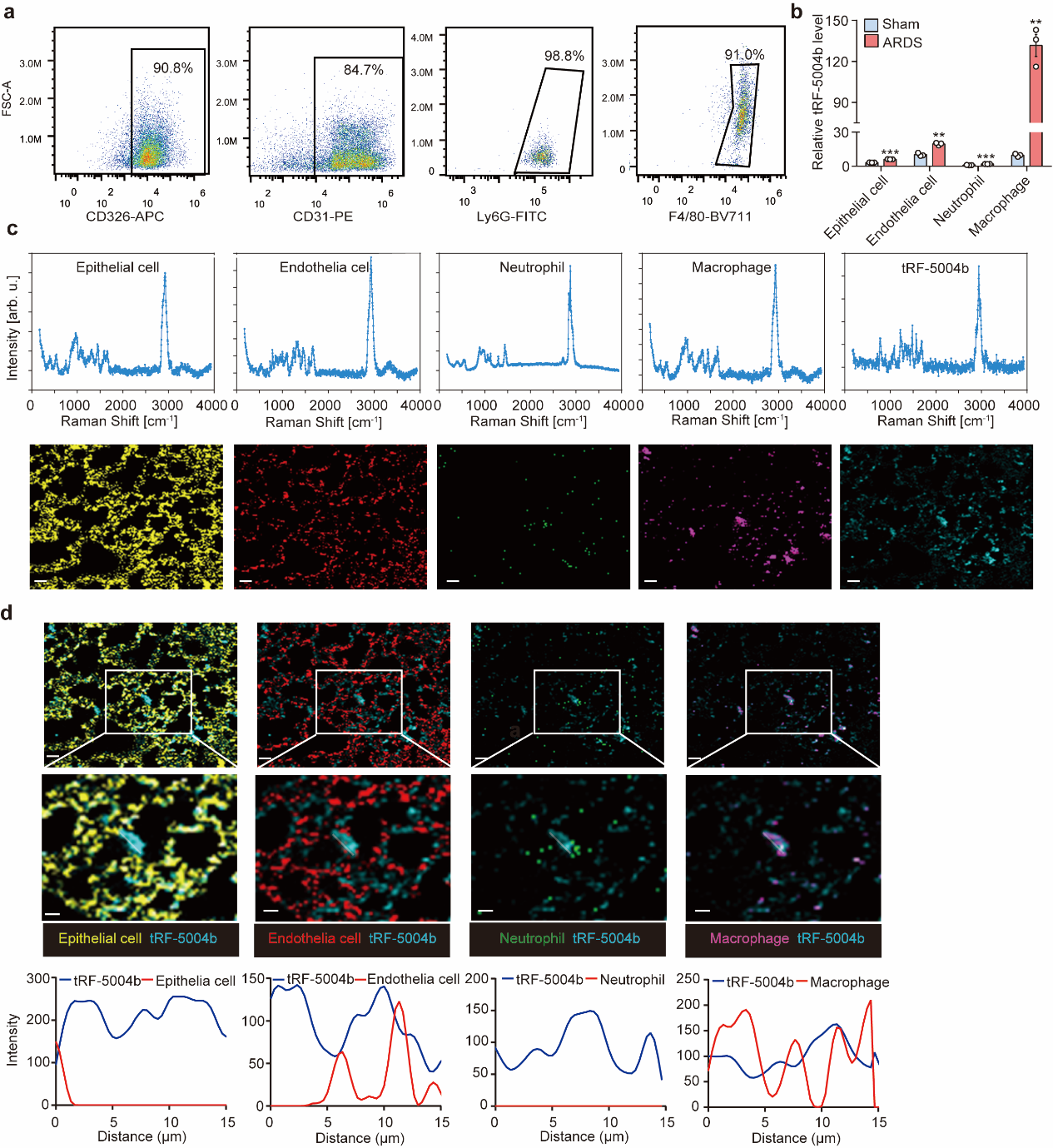


**Figure S16.** tRF-5004b is predominantly produced by macrophages in ARDS. a) The purity of sorted cell populations, including epithelial cells (Live/CD45-/CD326+), endothelial cells (ECs) (Live/CD45-/CD31+), neutrophils (Live/CD45+/Ly6G+), and macrophages (Live/CD45+/F4/80+), was assessed using flow cytometry. b**)** qRT-PCR results of the expression of tRF-5004b across sorted cell populations within lung tissue (n=3). c) Raman signals of the epithelial cell, ECs, neutrophil, macrophage and tRF-5004b reference standard, scale bar: 20 µm. d) Representative Raman imaging illustrates the co-localization of the four cell types and tRF-5004b within the lung tissue of ARDS mouse (n=1). Scale bars: 20 μm (wide ﬁeld) and 10 μm (magniﬁed spot). A line chart provides a positional analysis of the Raman signals. Statistics: unpaired two-tailed t-test or two-tailed Mann–Whitney U-test in (b). Data are represented as mean ± SEM. **p < 0.01, ***p < 0.001.


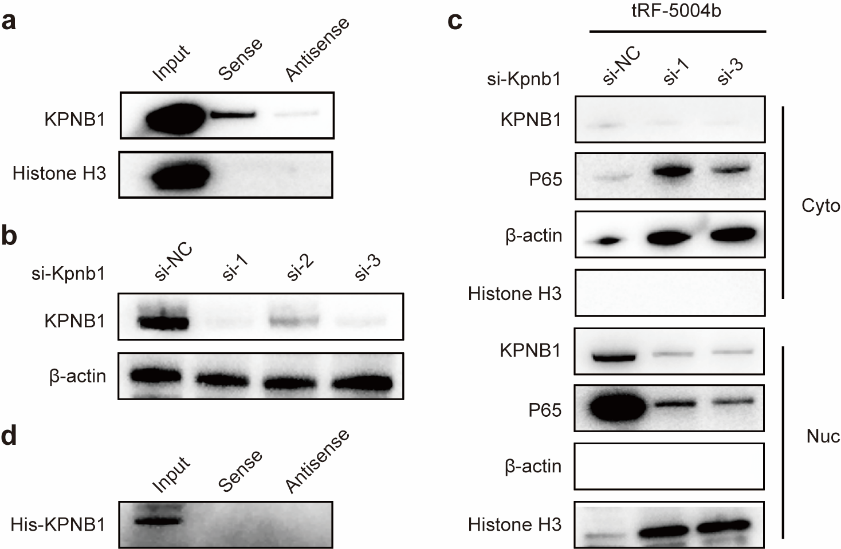


**Figure S17.** tRF-5004b is not directly bound to KPNB1. a) KPNB1 was pulled down by tRF-5004b in ECs, as determined by western blotting (n=1). b) KPNB1-knockdown efficiency of ECs at 24 h (n=1). c) Western blotting analysis of nuclear translocation of p65 in KPNB1-knockdown ECs or the NC-treated ECs stimulated by tRF-5004b-MSAPs for 12 h (n=3). d**)** In vitro RNA pull-down assay with purified recombinant His-AKPNB1 and tRF-5004b (n=3).


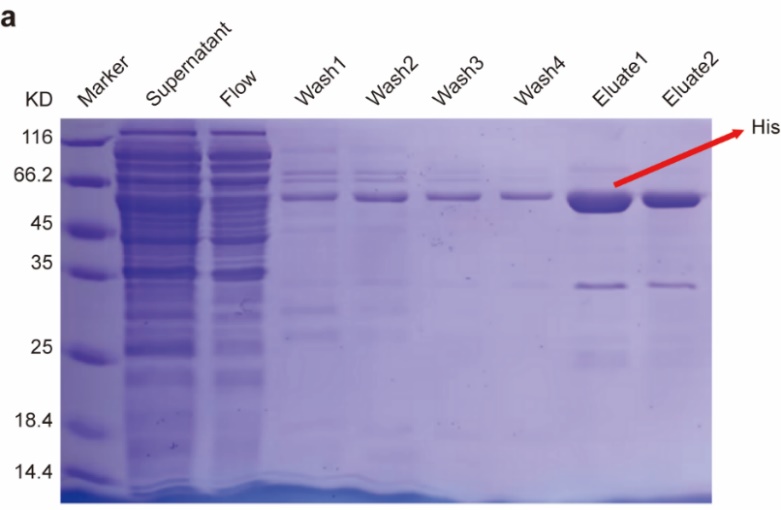


**Figure S18.** The recombinant protein His-KPNA2 in the supernatant was separated and purified successfully. a) The Coomassie Blue-stained gel shows the purified His-KPNA2 proteins.


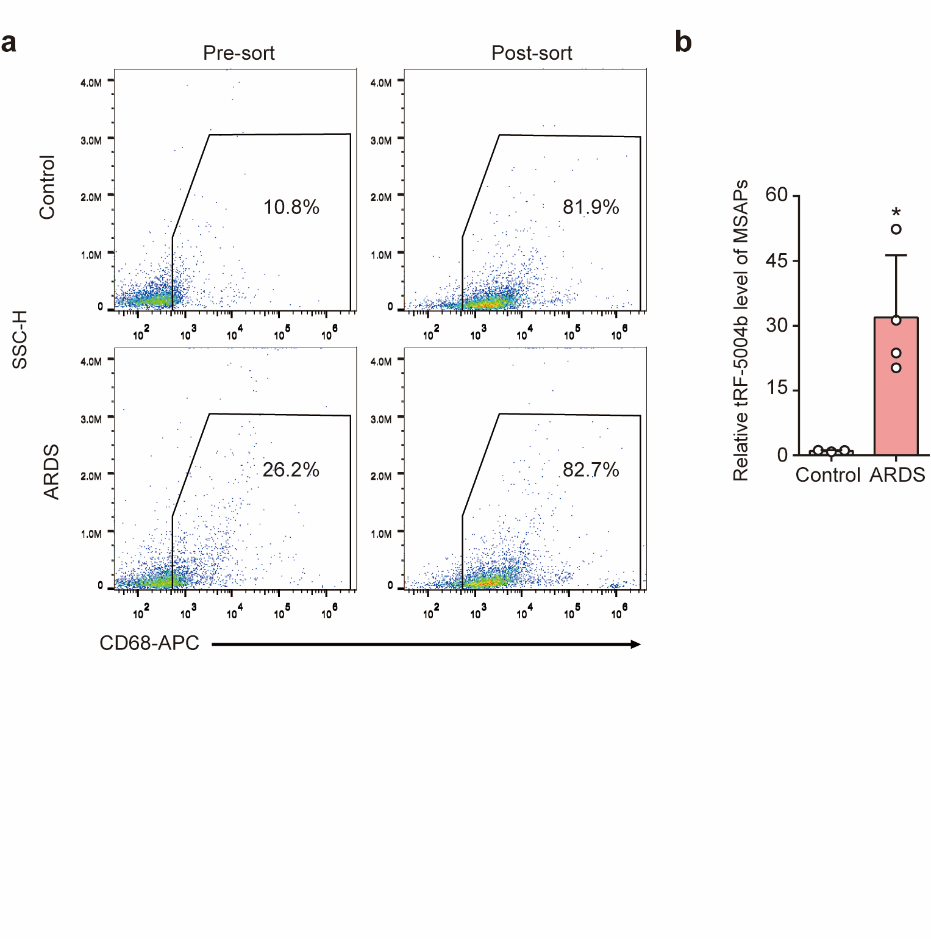


**Figure S19.** The tRF-5004b levels in BALF-MSAPs increased in ARDS patients compared to control group. a) Sorted MSAPs (CD68+) from control and ARDS were checked for purity by flow cytometry. b) The BALF-MSAP tRF-5004b level in Day 1 ARDS patients (n = 4) and controls (n = 3). Statistics: unpaired two-tailed t-test in (b). Data are represented as mean ± SEM. **p* < 0.05.


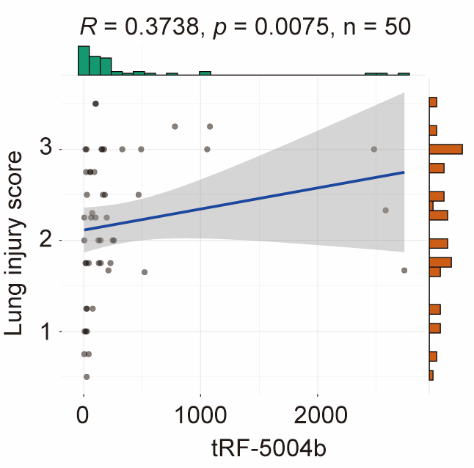


**Figure S20.** The tRF-5004b level was positively correlated with the lung injury score. Correlation of the lung injury score with the BALF-SAP tRF-5004b level (n=50). Statistics: Pearson’s correlation coeﬃcient analysis in (a). Data are represented as mean ± SEM.

**Table S1. Clinical characteristics of patients.**

| Parameters | Control (n=7) | ARDS (n=50) | *P value* |
| --- | --- | --- | --- |
| Age (year) | 55.14 (16.92) | 68.62 (12.84) | 0.0493 |
| Gender |  |  | 0.2886 |
| Male | 4 (57.1) | 38 (76.0) |  |
| Female | 3 (42.9) | 12 (24.0) |  |
| Cause of ARDS, n (%) | | | |
| Direct ARDS | 0 (0) | 33 (66.0) |  |
| Indirect ARDS | 0 (0) | 17 (34.0) |  |
| SOFA score | 2.14 (1.07) | 19.88(3.67) | < 0.0001 |
| APACHE II score | 8.57(4.08) | 20.94 (5.66) | < 0.0001 |
| Murray score | 0 (0) | 2.19 (0.77) | < 0.0001 |
| ICU stay (day) | 2.43(1.51) | 17.38(10.77) | < 0.0001 |

Categorical variables are expressed as n (%), and continuous variables are expressed as the mean (SD). *P* values less than 0.05 were considered statistically significant.

Abbreviations: ARDS, acute respiratory distress syndrome; SOFA, Sequential Organ Failure Assessment; APACHE, Acute Physiology and Chronic Health Evaluation; ICU, intensive care unit;

**Table S2. Antibodies used for flow cytometry**

| **Antibody** | **Company** | **Catalog #** | **Dilute** |
| --- | --- | --- | --- |
| APC conjugated Anti-mouse LC3 | Abcam | ab225383 | 1:100 |
| APC conjugated Anti-mouse ICAM1 | Biolegend | 116119 | 1:100 |
| PE conjugated Anti-mouse CD45 | Biolegend | 103106 | 1:100 |
| FITC conjugated Anti-mouse CD11b | Biolegend | 101206 | 1:100 |
| Percp-Cy5.5 conjugated Anti-mouse Ly6G | Biolegend | 127615 | 1:100 |
| FITC conjugated Anti-mouse VCAM1 | Biolegend | 105705 | 1:100 |
| PE conjugated Anti-mouse CD31 | BD | 553373 | 1:100 |
| PE conjugated Anti-human CD68 | Biolegend | 333810 | 1:100 |
| APC conjugated Anti-mouse CD326 | BD | 563478 | 1:100 |
| BV711 conjugated Anti-mouse F4/80 | BD | 565612 | 1:100 |
| FITC conjugated Anti-mouse Ly6G | BD | 561105 | 1:100 |
| Percp-Cy5.5 conjugated Anti-mouse CD45 | BD | 550994 | 1:100 |
| Viability Dye eFluor 510 | BD | 564406 | 1:100 |

**Table S3.** **Results of univariate logistic regression models for severe ARDS**

| **Characteristics** | **OR** | **95%CI** | | ***P* value** |
| --- | --- | --- | --- | --- |
|  |  | **lower** | **upper** |  |
| Gender | 0.758 | 0.127 | 4.519 | 0.761 |
| Age | 1.1 | 0.983 | 1.232 | 0.097 |
| tsRNA | 30.482 | 3.182 | 292.024 | 0.003 |
| IL-6 | 0.997 | 0.983 | 1.012 | 0.701 |
| TNF-α | 0.95 | 0.917 | 0.984 | 0.004 |
| TGF-β1 | 0.994 | 0.99 | 0.998 | 0.001 |
| IL-10 | 0.893 | 0.831 | 0.958 | 0.002 |
| WBC | 0.97 | 0.85 | 1.107 | 0.65 |
| N% | 0.973 | 0.927 | 1.022 | 0.277 |
| CRP | 1.006 | 0.996 | 1.015 | 0.265 |
| PCT | 0.955 | 0.854 | 1.068 | 0.419 |
| GAP | 0.898 | 0.7 | 1.152 | 0.397 |
| LAC | 1.171 | 0.907 | 1.513 | 0.226 |

Abbreviations: WBC, white blood cell; N%, neutrophil percentage; CRP, C-reactive protein; PCT, procalcitonin; GAP, Anion Gap; LAC, Lactic Acid;

**Table S4. Results of multiple step-wise logistic regression models for severe ARDS**

| **Characteristics** | **OR** | **95% CI** | | ***P* value** |
| --- | --- | --- | --- | --- |
|  |  | **lower** | **upper** |  |
| Age | 1.172 | 0.919 | 1.494 | 0.201 |
| tsRNA | 19.642 | 1.134 | 340.293 | 0.041 |
| TNF-α | 1.486 | 0.562 | 3.929 | 0.424 |
| TGF-β1 | 0.966 | 0.889 | 1.051 | 0.424 |
| IL-10 | 0.716 | 0.333 | 1.542 | 0.393 |

**Table S5. Results of the univariate Cox regression models for 28-day mortality**

| **Characteristics** | **HR** | **95% CI** | | ***P* value** |
| --- | --- | --- | --- | --- |
|  |  | **lower** | **upper** |  |
| Gender | 1.369 | 0.432 | 4.338 | 0.594 |
| Age | 1.038 | 0.986 | 1.093 | 0.155 |
| tsRNA | 3.414 | 1.496 | 7.79 | 0.004 |
| IL-6 | 1.006 | 0.996 | 1.015 | 0.226 |
| TNF-α | 0.979 | 0.959 | 0.999 | 0.044 |
| TGF-β1 | 0.997 | 0.995 | 0.999 | 0.002 |
| IL-10 | 0.962 | 0.928 | 0.996 | 0.031 |
| WBC | 0.873 | 0.788 | 0.968 | 0.01 |
| CRP | 1.007 | 1.001 | 1.014 | 0.028 |
| PCT | 1.037 | 1.004 | 1.071 | 0.026 |
| LAC | 1.161 | 1.019 | 1.322 | 0.025 |
| APACHEⅡ | 1.012 | 0.925 | 1.107 | 0.792 |
| SOFA | 1.234 | 1.05 | 1.45 | 0.011 |
| ALT | 1 | 0.995 | 1.005 | 0.881 |
| AST | 1 | 0.999 | 1.002 | 0.519 |
| BUN | 1.022 | 0.933 | 1.121 | 0.638 |
| Cr | 1 | 0.998 | 1.003 | 0.801 |
| P/F ratio | 0.999 | 0.993 | 1.006 | 0.814 |

Abbreviations: ALT, alanine aminotransferase; AST, aspartate transaminase; BUN, blood urea nitrogen; Cr, creatinine;

**Table S6.** **Results of the multiple step-wise Cox regression models of 28-day mortality**

| **Characteristics** | **HR** | **95% CI** | | ***P* value** |
| --- | --- | --- | --- | --- |
|  |  | **lower** | **upper** |  |
| tsRNA | 3.449 | 1.142 | 10.412 | 0.028 |
| TNF-α | 1.068 | 1.021 | 1.117 | 0.004 |
| TGF-β1 | 0.994 | 0.989 | 0.998 | 0.006 |
| SOFA | 0.994 | 0.989 | 0.998 | 0.006 |

**Table S7. Sequences of primers for qPCR**

| Prime | Sequence (5’ to 3’) |
| --- | --- |
| mouse-*Icam-1*-F | GGCAGCAGGTGGGCAAGAAC |
| mouse-*Icam-1*-R | CTGGCGGCTCAGTGTCTCATTC |
| mouse-*Vcam-1*-F | GAGGGTGGTGCTGTGACAATGAC |
| mouse-*Vcam-1*-R | GGGTGGCATTTCCTGAGAGAAGC |
| mouse-*Il-6*-F | ACCAGAGGAAATTTTCAATAGGC |
| mouse-*Il-6*-R | TGATGCACTTGCAGAAAACA |
| mouse-*Il-1β*-F | GGTCAAAGGTTTGGAAGCAG |
| mouse-Il-1β-R | TGTGAAATGCCACCTTTTGA |
| mouse-*Kpna2*-F | AATAGAACCTTTGATGAACCTCCTGAG |
| mouse-*Kpna2*-R | AGCCTCCACACTCTTCAATCATTATAC |
| mouse-*Xpot*-F | GAGGTTGAAGTAGCCGTGCG |
| mouse-*Xpot*-R | TCCTGATGTCACCAGCGTCC |
| mouse-*β-actin*-F | GTGACGTTGACATCCGTAAAGA |
| mouse-*β-actin*-R | GCCGGACTCATCGTACTCC |
| mouse-*Gapdh-*F | AGGTCGGTGTGAACGGATTTG |
| mouse-*Gapdh-*R | TGTAGACCATGTAGTTGAGGTCA |
| *U6*-F | CAGCACATATACTAAAATTGGAACG |
| U6-R | ACGAATTTGCGTGTCATCC |
| Gly-CCC-2-RT | GTCGTATCCAGTGCAGGGTCCGAGGTATTCGCACTGGATACGACGGCGAG |
| *Gly-CCC-2*-F | AAGCGACCGCATTGGTAGTTCA |
| *Gly-CCC-2*-R | ATCCAGTGCAGGGTCCGAGG |
| *Val-CAC-1*-RT | GTCGTATCCAGTGCAGGGTCCGAGGTATTCGCACTGGATACGACGGCGAG |
| *Val-CAC-1*-F | GTTTCCGTAGTGTAGTGGTTATCAC |
| *Val-CAC-1*-R | ATCCAGTGCAGGGTCCGAGG |
| *Gly-TCC-1*-RT | GTCGTATCCAGTGCAGGGTCCGAGGTATTCGCACTGGATACGACGGCAGC |
| *Gly-TCC-1*-F | GCGTTGGTGGTATAGTGGTGA |
| *Gly-TCC-1*-R | ATCCAGTGCAGGGTCCGAGG |
| *Gly-CCC-1*-RT | GTCGTATCCAGTGCAGGGTCCGAGGTATTCGCACTGGATACGACTCTTGC |
| *Gly-CCC-1*-F | AATAGTGCGCCGCTGGTGTAG |
| *Gly-CCC-1*-R | ATCCAGTGCAGGGTCCGAGG |
| *tRF-5004b*-RT | GTCGTATCCAGTGCAGGGTCCGAGGTATTCGCACTGGATACGACGGCGAG |
| *tRF-5004b*-F | CATTGGTGGTTCAGTGGTAGAATT |
| *tRF-5004b*-R | AGTGCAGGGTCCGAGGTATT |

**Table S8. Antibodies used for western blotting and immunofluorescence**

| **Antibody** | **Company** | **Catalog #** | **Dilute** |
| --- | --- | --- | --- |
| Rabbit anti-ICAM-1 | Abcam | ab222736 | 1:1000 |
| Rabbit anti-VCAM-1 | Abcam | ab134047 | 1:1000 |
| Mouse anti-P65 | CST | 6956S | 1:1000 |
| Rabbit anti-GAPDH | Starter | S0B0261 | 1:5000 |
| Rabbit anti-Histone H3 | Starter | S0B0079 | 1:1000 |
| Mouse anti-KPNB1 | Abcam | Ab2811 | 1:5000 |
| Rabbit anti-KPNA2 | Abcam | ab70160 | 1:1000 |
| Rabbit anti-LC3 | Abcam | ab182890 | 1:1000 |
| Mouse anti-FLAG | Sigma | F1804 | 1:2000 |
| Rabbit anti-HA | CST | 3724S | 1:1000 |
| Mouse anti-His | Proteintech | 66005-1-Ig | 1:2000 |
| Rabbit anti-β-actin | Starter | S0B0005 | 1:1000 |
| Rabbit anti-CD31 | Abcam | ab28364 | 1:200 |
| Rabbit anti-P65 | CST | 8242 | 1:200 |

**Table S9. siRNA and shRNA sequences used in this study**

| NO. | TargetSeq |
| --- | --- |
| NC | TTCTCCGAACGTGTCACGT |
| si-*KPNA2*-1 | GTTCAGCTTTCCGAGACTT |
| si-*KPNA2*-2 | CACTAAGAGCCATAGGGAA |
| si-*KPNA2*-3 | GTATACAAGGCCTCATTGA |
| si-*KPNB1*-1 | GGGAAGTCAAGAACTATGT |
| si-*KPNB1*-2 | CGACTCTTCAGAATGTTCT |
| si-*KPNB1*-3 | GTAGAAGCTAGGCCAATGA |
| si-*XPOT1*-1 | GGAGCAGTGCATTCCAAAT |
| si-*XPOT1*-2 | GCTTGTTCGAGATTGTAAA |
| si-*ALKBH3* | GGAGCAGCTTTGTAAGGAT |
| *sh-tRF-5004b* | GGCGAGAATTCTACCACTGAACCACCAATGC |

**Table S10. The sequence of mimics**

| **Name** | **Sequence (5’- 3’)** |
| --- | --- |
| NC | UUCUCCGAACGUGUCACGUTTACGUGACAC GUUCGGAGAATT |
| tRF-5004b | GCAUUGGUGGUUCAGUGGUAGAAUUCUCGCC |
| Gly-CCC-2 | GCATTGGTAGTTCAATGGTAGAATTCTCGCC |
| Val-CAC-1 | GTTTCCGTAGTGTAGTGGTTATCACGCTCGCC |
| Gly-TCC-1 | GCGTTGGTGGTATAGTGGTGAGCATAGCTGCC |
| Gly-CCC-1 | GCGCCGCTGGTGTAGTGGTATCATGCAAGA |

**Movie S1**

Real-time intravital imaging of endothelial- neutrophil adhesion of mice following intratracheal instillation with C-MSAPs.

**Movie S2**

Real-time intravital imaging of endothelial- neutrophil adhesion of mice following intratracheal instillation with L-MSAPs.
