# Supplementary figures and images for: tRF‐5004b Enriched Secretory Autophagosomes Induce Endothelial Cell Activation to Drive Acute Respiratory Distress Syndrome
Source: Adv Sci (Weinh). 2025 Jun 20;12(33):e03014. doi: 10.1002/advs.202503014 (PMC12412491; doi:10.1002/advs.202503014)

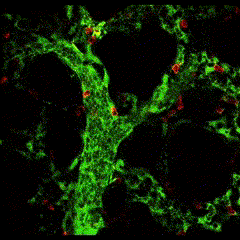

Supplement: Supplementary file 2 — Supplemental Movie 1 [file ADVS-12-e03014-s003.gif]

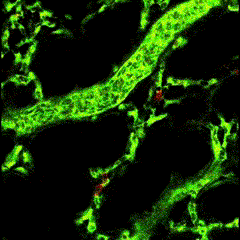

Supplement: Supplementary file 3 — Supplemental Movie 2 [file ADVS-12-e03014-s002.gif]
